# Potent Biological Activity of Fluorinated Derivatives of 2-Deoxy-d-Glucose in a Glioblastoma Model

**DOI:** 10.3390/biomedicines12102240

**Published:** 2024-10-01

**Authors:** Maja Sołtyka-Krajewska, Marcin Ziemniak, Anna Zawadzka-Kazimierczuk, Paulina Skrzypczyk, Ewelina Siwiak-Niedbalska, Anna Jaśkiewicz, Rafał Zieliński, Izabela Fokt, Stanisław Skóra, Wiktor Koźmiński, Krzysztof Woźniak, Waldemar Priebe, Beata Pająk-Tarnacka

**Affiliations:** 1 Department of Medical Biology, Kaczkowski Military Institute of Hygiene and Epidemiology, Kozielska 4, 01-163 Warsaw, Poland; maja.soltyka@gmail.com (M.S.-K.); ewelinasiwiak1@op.pl (E.S.-N.); ancpatrin@gmail.com (A.J.); 2 Biological and Chemical Research Centre, Department of Chemistry, University of Warsaw, Żwirki i Wigury 101, 02-089 Warsaw, Poland; mziemniak@chem.uw.edu.pl (M.Z.); anzaw@chem.uw.edu.pl (A.Z.-K.); pm.skrzypczyk@student.uw.edu.pl (P.S.); kozmin@chem.uw.edu.pl (W.K.); kwozniak@chem.uw.edu.pl (K.W.); 3 Department of Experimental Therapeutics, The University of Texas MD Anderson Cancer Center, 1901 East Rd., Houston, TX 77054, USA; rjzielinski@mdandreson.org (R.Z.); ifokt@mdanderson.org (I.F.); sskora@maandreson.org (S.S.); 4 WPD Pharmaceuticals, Żwirki i Wigury 101, 02-089 Warsaw, Poland

**Keywords:** glycolysis, 2-deoxy-d-glucose, halogenated derivatives, hexokinase activity, NMR spectroscopy, molecular docking, glycolysis inhibition, cytotoxic action

## Abstract

Background: One defining feature of various aggressive cancers, including glioblastoma multiforme (GBM), is glycolysis upregulation, making its inhibition a promising therapeutic approach. One promising compound is 2-deoxy-d-glucose (2-DG), a d-glucose analog with high clinical potential due to its ability to inhibit glycolysis. Upon uptake, 2-DG is phosphorylated by hexokinase to 2-DG-6-phosphate, which inhibits hexokinase and downstream glycolytic enzymes. Unfortunately, therapeutic use of 2-DG is limited by poor pharmacokinetics, suppressing its efficacy. Methods: To address these issues, we synthesized novel halogenated 2-DG analogs (2-FG, 2,2-diFG, 2-CG, and 2-BG) and evaluated their glycolytic inhibition in GBM cells. Our in vitro and computational studies suggest that these derivatives modulate hexokinase activity differently. Results: Fluorinated compounds show the most potent cytotoxic effects, indicated by the lowest IC_50_ values. These effects were more pronounced in hypoxic conditions. ^19^F NMR experiments and molecular docking confirmed that fluorinated derivatives bind hexokinase comparably to glucose. Enzymatic assays demonstrated that all halogenated derivatives are more effective HKII inhibitors than 2-DG, particularly through their 6-phosphates. By modifying the C-2 position with halogens, these compounds may overcome the poor pharmacokinetics of 2-DG. The modifications seem to enhance the stability and uptake of the compounds, making them effective at lower doses and over prolonged periods. Conclusions: This research has the potential to reshape the treatment landscape for GBM and possibly other cancers by offering a more targeted, effective, and metabolically focused therapeutic approach. The application of halogenated 2-DG analogs represents a promising advancement in cancer metabolism-targeted therapies, with the potential to overcome current treatment limitations.

## 1. Introduction

It has been observed that aggressive cancer types have an increased rate of glycolysis [1,2]. Pancreatic cancer and GBM are believed to primarily rely on the glycolytic metabolism of glucose [3,4,5]. This discovery has led to the idea that inhibiting glycolysis could be a promising strategy for treating GBM.

The current standard of care (SOC) for GBM treatment involves surgery, followed by radiotherapy and chemotherapy. For over two decades, the first-line chemotherapeutic has been temozolomide (TMZ), which is an alkylating agent. However, the clinical efficacy of TMZ is limited, resulting in a median survival of 14–15 months [6,7]. It is estimated that more than 50% of GBM patients do not respond to TMZ treatment due to an *O*-6-methylguanine-DNA methyltransferase (*mgmt*) promoter mutation. This leads to the upregulation of DNA repair mechanisms, which limits the effectiveness of TMZ [8,9]. Moreover, TMZ administration activates multidrug resistance proteins (MDR), which actively expel chemotherapeutic agents from the cell [10,11]. As recently reviewed, several drug candidates for GBM treatment exist, but only a few are in advanced stages (phase II or III) of clinical development [12]. Therefore, GBM chemotherapy is still an unmet medical need with significant clinical, sociological, and economic implications.

Reprogramming tumor cell metabolism from oxidative phosphorylation to aerobic glycolysis is a major strategy for promoting cell survival, progression, angiogenesis, and resistance to chemo- and radiotherapy [13,14]. While not all types of cancer rely solely on anaerobic glucose utilization, the most aggressive tumors, such as pancreatic ductal adenocarcinoma (PDAC) and glioblastoma multiforme (GBM), depend entirely on glycolysis. Thus, the old but simple concept of targeting cancer cells’ metabolism could be an effective strategy to introduce new drugs for GBM therapy [15,16].

The compound named 2-deoxy-D-glucose (2-DG) has been extensively studied due to its ability to inhibit glycolysis in cancer cells [17]. 2-DG is similar to glucose, but it lacks a hydroxyl group in the C-2 position [18]. It enters cells via glucose transporters (GLUTs) [19] and can cross the blood-brain barrier (BBB) to reach brain tissue [20]. Once inside the cell, 2-DG is rapidly converted to 2-DG-6-phosphate (2-DG-6P) via hexokinase (HK) [21,22]. However, 2-DG-6P cannot be processed further via phosphoglucose isomerase (PGI) and therefore accumulates inside the cells, inhibiting glycolysis [23]. In addition, 2-DG can also directly inhibit HK activity, further downregulating the glycolysis pathway [24,25]. This disruption in the glycolysis process leads to a disruption in ATP synthesis, resulting in the induction of autophagy and/or apoptosis [17]. Furthermore, 2-DG also interferes with mannose-dependent metabolism, leading to disruptions in protein maturation processes [17].

2-DG has been studied in various areas of cancer research, including GBM, due to its cytotoxic properties [2,26]. It has even been tested in clinical trials. However, despite being safe and well-tolerated, these clinical trials showed limited efficacy of 2-DG in cancer patients [17]. A phase 1 dose-escalation study of orally administered 2-DG in solid tumor patients, as reported by Raez et al. [27], revealed that the maximum tolerated dose (MTD) was 63 mg/kg. However, due to competition with innate glucose levels, which range from 7 to 10 mg/mL, the median maximum plasma concentration of only 0.116 mg/mL was achieved. These poor pharmacokinetic properties have limited the clinical introduction of 2-DG as an anticancer drug.

Prof. Priebe’s team developed new analogs of 2-DG to address its limitations and identify molecules that have similar biological efficacy but better pharmacokinetics. So far, only 2-fluoro-deoxy-d-glucose (2-FG) has been more widely tested as a potential anticancer agent, while for other halogen derivatives of 2-DG containing bromide, chloride, or iodide, only limited data are available or some derivatives have yet to be tested.

Hydrogen-to-halogen substitution in drug molecules can considerably impact binding to target proteins through various mechanisms. Halogens’ higher electronegativity alters electron distribution, affecting non-covalent interactions. Moreover, their larger size influences fit within binding pockets, potentially causing hindrance or enhancing complementarity. Increased lipophilicity improves membrane permeability and hydrophobic interactions. Halogens can form unique, directional halogen bonds, enhancing binding specificity and affinity. Halogenated compounds often exhibit increased stability, prolonging activity. The substitution can alter dipole moments and polarizability, affecting electrostatic and van der Waals interactions. Additionally, halogens can modulate pKa values of nearby groups, influencing charge states and ionic interactions [28].

The substitution of hydrogen atoms with halogens, particularly fluorine and chlorine, is a commonly used technique in medicinal chemistry to improve the pharmacological properties of small-molecule drugs. Such chemical modifications can enhance membrane permeation and metabolic stability as well as the affinity and specificity of interaction with their molecular targets [28,29,30]. For instance, a recent review of FDA-approved drugs in 2021 found that 14 out of 50 discussed compounds contained at least one halogen atom [31]. Moreover, some naturally occurring compounds containing halogen atoms, such as antibiotics like vancomycin [32] or halogenated metabolites produced by algae [33,34], have been introduced into clinical applications. Among all halogens, fluorine is most commonly used in the drug development process, primarily as a substituent of a hydrogen atom or methyl group (as CF_3_ group) [35]. In support of this, eight of the 14 previously mentioned FDA-approved drugs contained fluorine, while four contained chlorine and two contained a combination of the two. In contrast, none of the approved drugs contained bromine or iodine [31]. Fluorine substitution is also a common chemical modification in carbohydrates chemistry. A vast number of fluorinated sugars have been synthesized, including derivatives of common hexoses, such as glucose and mannose, as well as sialic acids or naturally existing deoxysugars [36].

Fluorine incorporation in carbohydrates influences their conformation at multiple levels. While fluorinated carbohydrates generally maintain the pyranose chair conformation, subtle changes can occur due to axial C–O and C–F bonds. In C-glycosides, fluorine substitution introduces *gauche* effects, stabilizing “non-natural” conformations through hyperconjugation. Notably, fluorination can restore conformational restrictions in C-glycosides, such as the *exo*-anomeric effect, typically present in non-fluorinated sugars. Due to its high ionization potential, fluorine is a poor hydrogen bond acceptor compared to atoms like oxygen, often leading to reduced hydrogen bond donation and weakened hydrogen bonding networks typical of natural sugars. While intramolecular hydrogen bonding involving fluorine is possible, it is generally weaker than traditional hydrogen bonds. Additionally, the hydrophobic nature of fluorine can induce the incorporation of extra water molecules in binding sites, affecting the energetics of protein-sugar interactions [37].

Fluorinated carbohydrates have proven valuable in biochemistry and cell biology research. These compounds have been used to study membrane transport mechanisms, with compounds like 6-deoxy-6-D-fluorofructose providing insights into GLUT2 and GLUT5-mediated transport [38]. Another compound, 6-deoxy-6-fluoro-D-galactose, was applied to study the biosynthesis of galactoproteins in L1210 leukemia cells [39]. The presence of ^19^F atoms enables ^19^F NMR spectroscopy, which was applied to investigate sugar-lectin interactions, among others [40]. Notably, ^19^F-STD NMR has enhanced sensitivity in detecting binding events between Con A and fluorinated glucose derivatives [41]. Methods that rely on *R*_2_ relaxation were also applied to study lectin-carbohydrate interactions [42]. Fluorinated glucose analogs mimic natural glucose, allowing to study enzyme interactions and the importance of specific OH groups in binding and metabolic processes [43]. In PET imaging, 2-FDG’s structural similarity to glucose enables visualization and quantification of glucose uptake in tissues. Additionally, fluorinated hexoses such as 3-deoxy-3-fluroglucose display varying effects on glycolytic enzymes, demonstrating their potential for selective enzyme inhibition [44]. Such ability presents opportunities for their use in the treatment of diseases, including viral infections and cancers. For instance, a series of l-fucose derivatives are also considered to be promising anticancer drugs, especially 6,6-difluoro-l-fucose, showing inhibitory effects on cell proliferation and angiogenesis [45].

In our previous research, we conducted a comprehensive analysis of the physical and chemical properties of halogen analogs of 2-DG using X-ray crystallography and NMR spectroscopy in solution [46,47]. Our studies focused on the non-covalent interactions in the crystalline state and the conformational equilibria in the aqueous solution. Despite H-to-halogen substitution, the conformation of their pyranose rings remains mostly unchanged. By gaining a better understanding of the structural chemistry of 2-DG analogs, we can predict their potential biological properties. It is worth noting that the potential polymorphism of the 2-DG derivatives’ crystals may have significant medical implications, as different crystal polymorphs may show distinct bioavailability or chemical stability, particularly when administered orally.

There is only limited research on the biochemical and physiological properties of halogenated analogs of 2-DG, and several compounds, namely 2-deoxy-2-fluoro-d-glucopyranose (2-FG), 2-deoxy-2-chloro-d-glucopyranose (2-CG), and 2-bromo-2-deoxy-d-glucopyranose (2-BG), were ever studied. It was found that their ability to inhibit glycolysis in osteosarcoma cells followed the order: 2-FG > 2-CG > 2-BG, with 2-FG showing the strongest inhibition; moreover, 2-FG displayed higher cytotoxicity than 2-DG. The computational studies on their interactions with hexokinase I showed the following order of binding affinity: 2-FG > 2-DG > 2-CG > 2-BG. d-glucose, the natural substrate, had the highest binding affinity to hexokinase [48]. In addition, a different study shows that 2-DG and 2-FG effectively inhibit glycolysis and disrupt the energy metabolism of *Plasmodium falciparum*, validating glycolysis as a viable drug target for antimalarial therapy [49].

Herein we present the in vitro data that demonstrates the biological activity of different halogen 2-DG derivatives, namely 2-deoxy-2-fluoro-d-glucopyranose (2-FG), 2-deoxy-2-chloro-d-glucopyranose (2-CG), 2-bromo-2-deoxy-d-glucopyranose (2-BG), and 2-deoxy-2,2-difluoro-d-glucopyranose (2,2-diFG). Specifically, we aimed to assess the cytotoxic effects of these analogs compared to 2-DG and investigate their impact on glycolysis by measuring cell viability, proliferation, protein synthesis, and lactate production under normoxic and hypoxic conditions. Next, we aimed to explore the binding interactions of these analogs with hexokinase via ^19^F NMR and molecular docking studies and to evaluate their enzymatic inhibition of hexokinase (HKII) and their potential to overcome the pharmacokinetic limitations of 2-DG. These objectives are central to developing a more effective and metabolically targeted treatment strategy for GBM and potentially other cancers.

In contrast to earlier studies on halogenated 2-DG analogs, more focused on cellular response, we employed a multifaced strategy also involving biochemical and computational methods in order to quantify both their binding affinities and intermolecular interactions with hexokinase II. Our findings showed that the fluorinated 2-DG analogs, 2-FG and 2,2-diFG, were the most potent cytotoxic agents, while 2-BG and 2-CG were practically ineffective. Based on the results of our biochemical assays and computational studies, we concluded that the observed differences in the compounds’ properties were due to their various ability to interact with and modulate HK activity.

We believe that our research on halogenated 2-DG analogs significantly contributes to the field of metabolic-targeted molecules in several essential ways. The fluorinated derivatives have shown enhanced potency compared to the original compound, marking a significant leap in cancer research. Furthermore, using computational and experimental approaches, our study provides detailed insights into the molecular interactions between these analogs and hexokinase, a key glycolytic enzyme. By demonstrating improved pharmacokinetics and efficacy at lower doses, our work suggests that these halogenated derivatives could serve as more effective alternatives to 2-DG, overcoming limitations such as short half-life, and low bioavailability. The findings open up new opportunities for targeted therapies against GBM and other glycolysis-dependent cancers, particularly in overcoming challenges like tumor hypoxia and treatment resistance.

## 2. Materials and Methods

In this study, we aimed to validate the biological potential of halogenated 2-DG derivatives in a glioblastoma model and assess the molecular mechanisms of these effects. To accomplish this, we utilized a combination of biological and physicochemical methods. The following section provides detailed information on the specific materials, protocols, and experimental approaches used to ensure the reliability and reproducibility of our results.

### 2.1. Reagents

**Figure 1:**
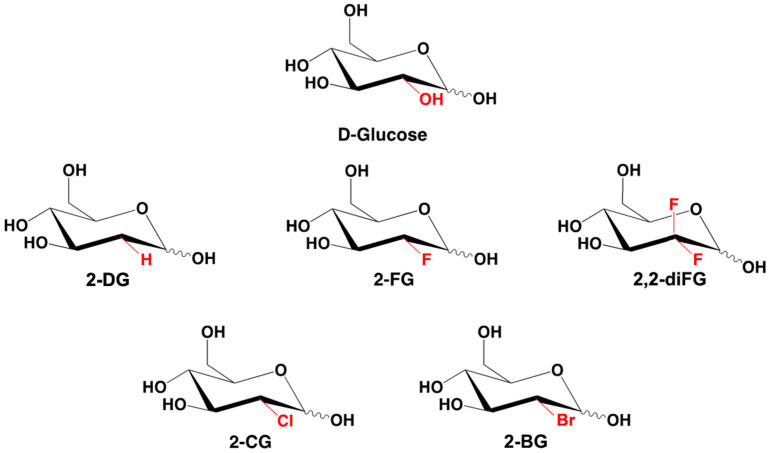
Chemical structures of d-glucose, 2-DG, and its halogenated derivatives: 2-FG, 2,2-diFG, 2-BG, 2-CG.

All chemical compounds and reagents used in this study were of analytical grade. 2-deoxy-2-fluoro-d-glucose (2-FG), 2-deoxy-2,2-difluoro-d-glucose (2,2-diFG), 2-bromo-2-deoxy-d-glucose (2-BG), and 2-chloro-2-deoxy-d-glucose (2-CG) were synthesized at the MD Anderson Cancer Institute by Waldemar Priebe’s group [50]. Their structures and purity were confirmed using NMR spectroscopy, high-resolution MS, and thin layer chromatography and compared with the literature data [50]. The chemical structures of synthesized derivatives are presented in Figure 1.

2-Deoxy-d-glucose (2-DG), phenazine methosulfate (PMS), dimethyl sulfoxide (DMSO), bovine serum albumin (BSA), cycloheximide (CHX), acetic acid, trichloroacetic acid (TCA), sulphorodamine B (SRB), chloroquine (CQ), rhodamine 123 (Rho123), dimethyloxalyglycine (DMOG), Lactate Assay Kit, and bromodeoxyuridine (BrdU) Cell Proliferation Kit were purchased from Sigma Aldrich (Saint Louis, MO, USA). 3-(4,5-dimethylthiazol-2-yl)-5-(3-carboxymethoxyphenyl)-2-(4-sulfophenyl)-2H tetrazolium inner salt (MTS) was purchased from Promega (Madison, WI, USA). Caspase Apoptosis Colorimetric Assay was purchased from G-Biosciences (Saint Louis, MO, USA) and Hexokinase Assay Kit from Abcam (Cambridge, UK).

### 2.2. Biological Analysis

#### 2.2.1. Cell Culturing

The U-251 and U-87 human glioblastoma multiforme cell lines were obtained from the European Collection of Authenticated Cell Cultures (ECACC, Salisbury, Wiltshire, UK). The cells were grown in Dulbecco’s Modified Eagle’s Medium (DMEM) containing either low glucose [1 g/L for U-87] or high glucose [4.5 g/L for U-251], along with 10% (*v*/*v*) heat-inactivated fetal bovine serum (FBS) (Biowest, Riverside, MO, USA), Amphotericin B (Thermo Fisher Scientific, Waltham, MA, USA) [1 μg/mL], Penicillin/Streptomycin (Life Technologies/Thermo Fisher Scientific, Waltham, MA, USA) [50 IU/mL/50 μg/mL], and Gentamicin sulfate (Sigma-Aldrich, Saint Louis, MO, USA) [20 μg/mL] at 37 °C and 5% CO_2_. Once the cell culture reached 75–80% confluence, the cells were seeded into 96-well plates or Petri dishes, depending on the experimental protocol. For viability, protein synthesis, and proliferation assays, cells were seeded in 96-well plates at 1.5 × 10^4^/mL. The cells were then treated with various concentrations of experimental compounds for 72 h. The following concentrations of 2-DG and its analogs were used: 2-DG [0.5–20 mM], 2-FG [1–10 mM], 2-IG [0.5–20 mM], 2-CG [5–40 mM], 2-BG [5–40 mM], and 2,2-diFG [0.5–15 mM]. The IC_50_ concentration was calculated for each compound using the following formula:

$${IC}_{50}=\frac{{\left[Treatment\right]}_{50}}{\left[Control\right]}$$

where: [Treatment]_50_ is the concentration of the drug at which 50% of cell viability is inhibited compared to untreated control cells. Calculations were made using GraphPad Prism™ version 8.0 software (GraphPad Software Inc., San Diego, CA, USA).

Cycloheximide (CHX), a protein synthesis inhibitor, was used as a positive control to induce cell apoptosis. Chloroquine (CQ), which inhibits autophagosome-lysosome fusion, was used to assess the induction of autophagy in treated cells. Prolyl hydroxylases inhibitor (DMOG) [50 μM] and rhodamine 123 (Rho123) [0.25 μM] were used to create hypoxia-mimicking conditions necessary to assess the influence of oxygen concentration on GBM cells’ sensitivity to the analyzed compounds. Both compounds were added to the cell medium 4 h before the treatment and remained until the end of the experiment. The expression of HIF-1α and its downstream proteins, lactate dehydrogenase A (LDHA) and pyruvate dehydrogenase kinase (PDK1), were assessed using the Western blot method.

#### 2.2.2. Cell Viability Determination

Cellular viability was determined by the MTS assay, which allows for assess mitochondrial dehydrogenase activity. Treated cells were incubated with 20 µL/well MTS solution (Promega, Madison, WI, USA) for 1 h at 37 °C. Mitochondrial dehydrogenases convert tetrazolium inner salt into insoluble purple formazan. Absorbance measurement was performed at 490 nm using a Synergy H1 multi-plate reader (BioTek, Winooski, VT, USA).

#### 2.2.3. Cell Proliferation Determination

Cell proliferation intensity was evaluated after exposure to various compounds was assessed using the bromodeoxyuridine (BrdU) Cell Proliferation Assay Kit (Sigma-Aldrich, Saint Louis, MO, USA). U-87 and U-251 cells were seeded at 1000 cells/well in a 96-well plate. Next, 20 µL of diluted BrdU solution was added to the cells and incubated for the last 24 h. Following this, cells were washed with phosphate-buffered saline (PBS) and fixed in fixing solution for 30 min. Then, they were incubated for 1.5 h at room temperature (RT) with mouse anti-BrdU-antibody, washed three times with PBS, and incubated with anti-mouse horseradish peroxidase (HRP) IgG (1:2000) (0.5 h, RT). After that, tetramethylbenzidine (TMB, HRP substrate) was added at RT for 30 min, and the reaction was halted by adding Stop Solution. Finally, the absorbance was determined at 450 nm with a Synergy H1 multi-plate reader (BioTek, Winooski, VT, USA).

#### 2.2.4. Protein Synthesis Determination

The amount of cellular content was determined using the sulphorodamine B (SRB) assay from Sigma Aldrich (Saint Louis, MO, USA). This method was optimized for the toxicity screening by Orellana and Kasinski [51]. At the end of the experiment, cells were fixed with 10% (*w*/*v*) trichloroacetic acid for 30 min at 4 °C, washed with water 4 times, and then stained with 0.04% SRB. The protein-bound dye was dissolved in a 10 mM Tris base solution (pH 10.5), and the absorbance was measured at 510 nm using a Synergy H1 multi-plate reader (BioTek, Winooski, VT, USA).

#### 2.2.5. Whole-Cell Lysates Preparation

Cells were seeded in a 60 or 100 mm diameter culture Petri dish. At the end of the experiment, 1 mL of ice-cold PBS was added, and the cells were scraped. They were then collected by centrifugation (4 °C, 15 min, 10,000× *g*). For lysing the cell pellet, we used 1 mL of radioimmunoprecipitation assay buffer (RIPA buffer), which is a mixture of 1x PBS, 10 mL/L, 5 g/L sodium deoxycholate, Igepal CA-630, and 1 g/L SDS. We also supplemented it with 10 μg/mL of aprotinin, 0.4 mM phenylmethylsulfonyl fluoride (PMSF), and 10 μg/mL of sodium orthovanadate (Sigma-Aldrich, St. Louis, MO, USA). We carried out repetitive triturating using a syringe (0.6 mm diameter) to break the cells. The cell lysate was left on ice (4 °C) for 30 min and then centrifugated for 5 min (4 °C, 10,000× *g*). Such obtained solution was transferred to fresh Eppendorf tubes and stored at −80 °C.

#### 2.2.6. Lactate Synthesis Assessment

The intensity of glycolysis can be measured by evaluating the levels of intracellular and extracellular lactate. To do this, a colorimetric test called the Lactate Assay Kit (Sigma-Aldrich, St. Louis, MO, USA), was used per the manufacturer’s protocol. At the end of the experiment, cells were washed with ice-cold PBS, scrapped off the 60 mm Petri dishes, and collected by centrifugation (4 °C, 6 min, 10,000× *g*). The pellets were then resuspended in 500 µL of Lactate Assay Buffer, transferred to Pierce Protein Concentrators with polyethersulfone (PES) 10 kDa (Thermo Fisher Scientific Invitrogen, Waltham, MA, USA), and centrifuged again (4 °C, 15 min, 8000× *g*) to remove intracellular lactate dehydrogenase. Next, 50 µL of supernatant (culture medium) was diluted and transferred in triplicates to a 96-well plate. 50 µL of Master Reaction Mix (46 μL Lactate Assay Buffer + 2 μL Lactate Enzyme Mix + 2 μL Lactate Probe) was added to each well, and plates were then incubated for 30 min in the dark. Finally, absorbance was determined at 570 nm using a Synergy H1 multi-plate reader (BioTek, Winooski, VT, USA).

#### 2.2.7. Hexokinase Activity Assay

The Hexokinase II Inhibitor Screening KIT from Abcam, Cambridge, UK, was used to evaluate the inhibition of HKII activity. The assay relied on the ability of HKII to phosphorylate glucose to glucose-6-phosphate, which is then oxidized by glucose-6-phosphate dehydrogenase, leading to the formation of the NADH. This product reduces the colorless probe to a colored product with an absorbance maximum at 450 nm. To determine the inhibition of HKII activity by tested compounds, different concentrations were prepared: 50 mM; 40 mM; 25 mM; 5 mM; 0.5 mM; 0.05 mM; and 0.005 mM. Positive control [50 µM of bromopyruvatic acid] and enzyme control were also prepared. All probes (50 µL) were loaded into a 96-well plate, 5 µL of HKII enzyme was added to each well, and incubated for 5 min at RT. After 5 min, 45 µL of reaction substrate mix (29 µL of buffer, 10 µL of HKII substrate, 2 µL of coenzyme, 2 µL of converter, and 2 µL of developer) was added into the wells. HKII activity was determined by a kinetic measurement for 40 min at 450 nm.

### 2.3. Physicochemical Analysis

#### 2.3.1. Expression and Purification of HKII Protein

The synthetic gene of HKII, codon-optimized to express in *E. coli*, was prepared by Thermo Fisher. It was then expressed in an *E. coli* BL21 D3 strain using the pMCSG7 expression plasmid. Molecular cloning was carried out using the NEBuilder HiFi DNA Assembly kit (New England Biolabs, Ipswich, MA, USA). To remove the first 16 aa from the N-terminal, a Q5 Site-Directed Mutagenesis Kit (New England Biolabs, Ipswich, MA, USA) was used. HKII protein expression was performed in LB medium at 37 °C. When the optical density of the culture reached 0.6, an overnight expression (at 16 °C) was induced by 0.1 M IPTG. The cells were harvested by centrifugation (4 °C, 6000× *g*, 20 min). The cells were resuspended in a buffer containing 15 mM Tris-HCl, 50 mM glucose, 0.5% Triton X-100, 9% glycerol, 0.2 mM PMSF, 2 mM BME, and several mg/L lysozyme powders. A total of 40 mL of the buffer was used per 1 L of bacterial culture. The cells were disrupted by sonication (2 s pulse, t = 10 min, 40% amplitude) and then centrifuged (4 °C, 26,500× *g*, 20 min). The supernatant was filtered and mixed with washed ProBondTM Nickel-Chelating Resin from Life Technologies/Thermo Fisher Scientific, Waltham, MA, USA. The resin with supernatant was equilibrated for 1 h at 4 °C, loaded onto a gravity column, washed with lysis buffer, and subsequently eluted with the lysis buffer with 300 mM imidazole. The eluate was concentrated using centrifugal devices from Pall Corporation, New York, NY, USA. The lysis buffer was exchanged for FPLC buffer containing 500 mM NaCl, 0.5 mM TCEP, 25 mM phosphate, pH 8.0. Upon concentration, the protein solution was subjected to FPLC-SEC purification with a GE Superdex 200 16/60 column. Fractions of the HKII protein were combined and concentrated to ~5 mg/mL in FPLC buffer. The concentration of HKII was determined from UV/VIS spectrophotometry.

#### 2.3.2. NMR Titrations and Determination of *K_d_*

All NMR experiments were conducted at 25 °C using a OneNMR room-temperature probe on a 600 MHz Bruker spectrometer.

##### Determination of *K_d_* of Fluorinated Ligands (2-FG and 2,2-diFG)

For each ligand, nine samples were prepared. One of these samples contained only the ligand, with a concentration of 2 mM. The other eight samples contained both the HKII protein, with a concentration of 0.01 mM and the ligand at the following concentrations: 0.25, 0.5, 0.8, 1.0, 1.5, 2.5, 4.0, and 6.0 mM. To determine the observed transverse relaxation rates, the fluorine-detected CPMG experiment was performed for each sample. In the case of samples containing the protein, the relaxation rates of the free and bound forms were averaged and weighted by populations of the two forms [52]. The observed relaxation rates were determined separately for α and β anomers, which were observed as separate signals on NMR spectra, as the anomerization occurs in a slow-exchange regime. The relaxation delays used for the relaxation measurements were 0.004, 0.016, 0.064, 0.128, 0.256, 0.512, 1.024, and 2.048 s. Non-linear data fitting was performed to determine the dependence of the observed relaxation rates on the ligand concentration, using the formulas S14 and S15 (see the ESI for details). All calculations were performed using homemade Python 3 scripts. The *symfit 0.5.6* package was used for non-linear fitting, using the *fit* function and default parameters [53].

##### Investigation of Interaction of Non-Fluorinated Ligands (2-BG, 2-CG, 2-DG) with HKII Protein

Nine samples were prepared for each ligand. All the samples had 0.01 mM of HKII protein, 2 mM of 2-FG, and a certain amount of non-fluorinated ligands. The concentration of this ligand in consecutive samples was 0.25, 0.5, 1, 1.5, 2.5, 4, 6, 8, and 10 mM. The fluorine-detected CPMG experiment was performed for each sample to determine the observed transverse relaxation rates of the fluorine-containing ligand at different concentrations of non-fluorinated ligand. However, due to theoretical difficulties described in the Supplementary Materials (Equations (S1) and (S2)), it was not possible to determine the dissociation constants for the HKII interaction with non-fluorinated ligands. Therefore, only qualitative results are provided.

#### 2.3.3. Molecular Docking and Binding Analysis

A structural model of HKII complexed by D-glucose and D-glucose-6-phosphate (G-6-P) is available in the PDB database (entry 2NZT). We selected the 2NTZ structure for molecular docking since it is the only human hexokinase II structure available in the PDB database. Other similar entries are structures of yeast hexokinase II or other isoforms of human hexokinases. Since 2NTZ has glucose as a ligand, it allowed to compare docking results of glucose to the experimental findings (initial assessment of the reliability of the docking method). This structural model was used as a template for molecular docking, after being truncated to its C-terminal domain. All non-protein fragments, such as ligands, cofactors, solvent molecules, etc., were removed using the *ChimeraX 1.5* program [54].

To perform molecular docking, the Dynamic Modified Restricted Tournament method [55] was used, which was implemented in DocThor web server (https://dockthor.lncc.br; accessed on 2 September 2024) [56]. The grid was either centered at the approximate center of mass of the innate *Glc* molecule (grid dimensions 12 × 12 × 12 Å, discretization step 0.25 Å) or using blind docking (grid dimensions 40 × 40 × 40 Å, discretization step 0.42 Å). The same setting of the genetic algorithm was used in both cases (10^6^ evaluations, population size = 750, number of runs = 24, number of poses per run = 10). The MLR DockTScore empirical scoring function was used to estimate both binding affinities and internal energies of the docked ligands [57]. This scoring function combines physics-based terms with machine-learning algorithms. All visualizations and analyses of ligand-protein interactions were prepared using ChimeraX. Hirshfeld Surfaces (HSs) and two-dimensional fingerprint plots were calculated using *Crystal Explorer 21* [58], using experimental atomic coordinates adjusted by *Crystal Explorer.* Input.cif files were prepared using ChimeraX and CCDC Mercury 4.0 software [59].

### 2.4. Statistical Analysis

All statistical analyses were conducted using GraphPad Prism™ version 8.0 software (GraphPad Software Inc., San Diego, CA, USA). For experiments comparing multiple groups, one-way ANOVA was used, followed by Tukey’s multiple comparisons test. For experiments analyzing both time and treatment effects, a two-way ANOVA was applied, followed by Bonferroni’s post-hoc test to adjust for multiple comparisons. Results for all treatment groups are expressed as a percentage of the control group. The percentage of control (% of control) was calculated using the following formula:

$$\%\;of\;control=\left(\frac{mean\;of\;treatment\;group}{mean\;of\;control\;group}\right)\times 100$$

This formula ensures that all treatments are normalized relative to the untreated control group, allowing for standardized comparisons across different experimental conditions. Data are presented as mean ± standard error of the mean (S.E.M.) from at least three independent experiments to ensure reproducibility. Statistical significance was defined as follows: * *p* < 0.05, ** *p* < 0.01, *** *p* < 0.001, with lower *p*-values indicating stronger significance. Error bars on the graphs represent S.E.M., showing the variability between independent experiments. All experiments included proper controls to ensure the reliability and validity of the data.

## 3. Results

In the ongoing search for more effective treatments for glioblastoma multiforme (GBM), evaluating novel therapeutic agents with enhanced specificity and efficacy is critical. This section details our findings on the cytotoxic effects of halogenated 2-deoxy-d-glucose (2-DG) analogs. Our approach combines both experimental and computational studies to assess the impact of these analogs on GBM cells. We aim to identify which compounds exhibit the greatest potential for glycolysis inhibition and therapeutic efficacy by analyzing cell viability, proliferation, and metabolic activity as well as their binding affinity via ^19^F NMR relaxation experiments. Additionally, computational studies provide insights into the binding interactions between these analogs and HKII. The following subsection presents the results of our investigations, highlighting the potent cytotoxic effects observed with the fluorinated 2-DG derivatives and their implications for GBM treatment.

### 3.1. Fluorinated 2-DG Derivatives Exert Potent Cytotoxic Effects on GBM Cells

**Figure 2:**
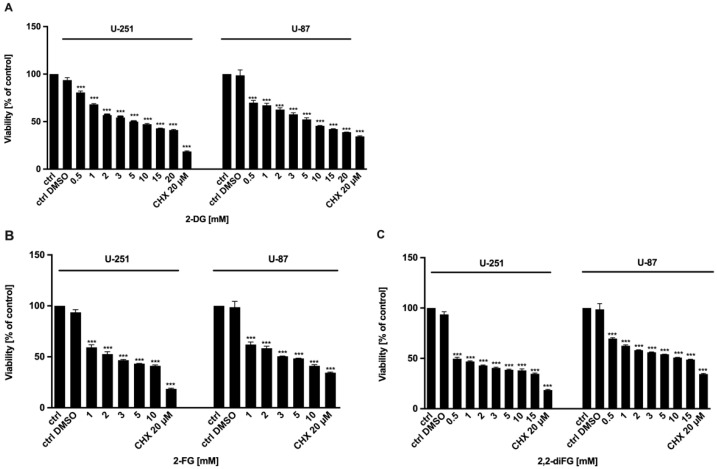
Viability of U-87 and U-251 cells after 72 h treatment with various concentrations of (**A**) 2-DG [0.5–20 mM] and its halogen derivatives: (**B**) 2-FG [1–10 mM], (**C**) 2,2-diFG [0.5–15 mM]. Protein synthesis inhibitor CHX [20 μM] was used as a positive cytotoxic control. Significant differences between the treatment and control means are indicated by *** *p* < 0.001.

**Figure 3:**
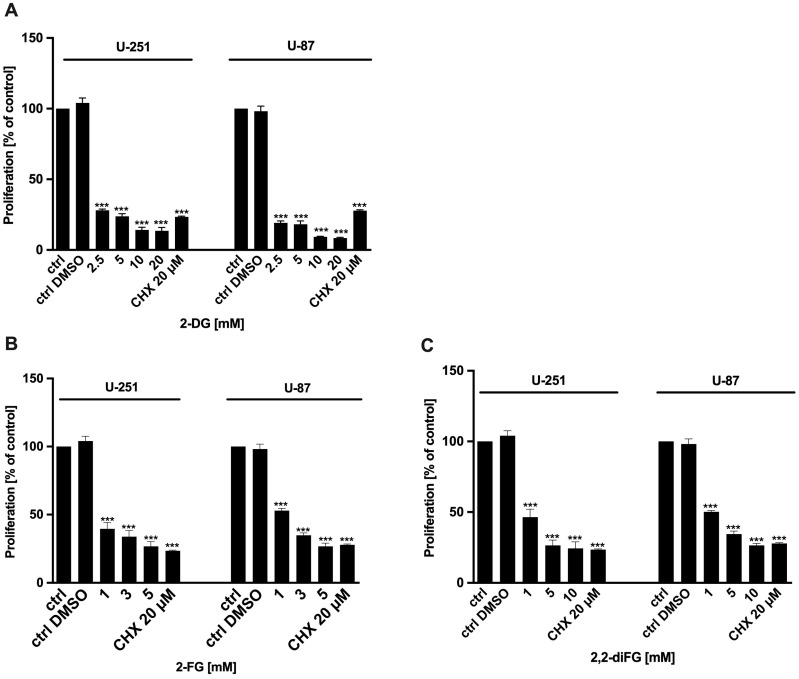
Proliferation of U-87 and U-251 cells after 72 h treatment with various concentrations of (**A**) 2-DG [2.5–20 mM] and its halogen derivatives: (**B**) 2-FG [1–5 mM], (**C**) 2,2-diFG [1–10 mM]. Protein synthesis inhibitor CHX [20 μM] was used as a positive cytotoxic control. Significant differences between the treatment and control means are indicated by *** *p* < 0.001.

**Figure 4:**
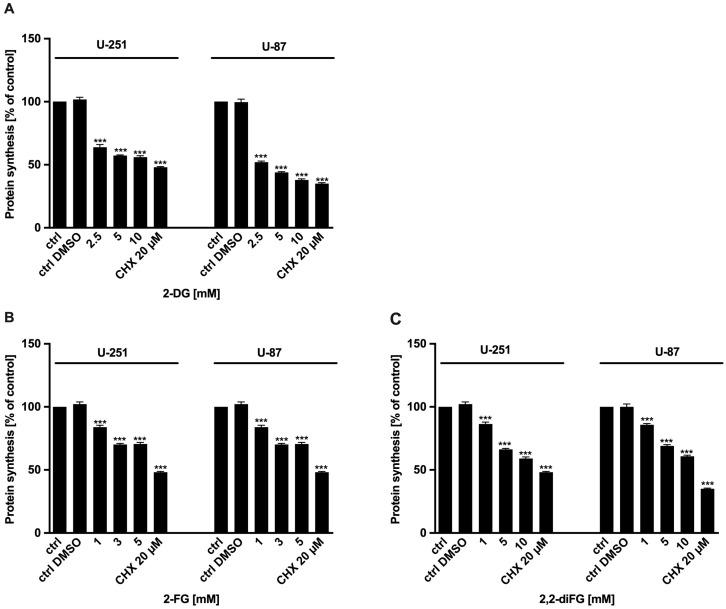
Protein synthesis of U-87 and U-251 cells after 72 h treatment with various concentrations of (**A**) 2-DG [2.5–10 mM] and its halogen derivatives: (**B**) 2-FG [1–5 mM], (**C**) 2,2-diFG [1–10 mM]. Protein synthesis inhibitor CHX [20 μM] was used as a positive cytotoxic control. Significant differences between the treatment and control means are indicated by *** *p* < 0.001.

To evaluate the cytotoxic potential of halogen-substituted 2-DG derivatives, we conducted a series of experiments to examine cell viability (Figure 2—2-DG, 2-FG, 2,2-diFG; Figure S1—2-FG, 2,2-diFG, 2-BG, 2-CG), proliferation (Figure 3—2-DG, 2-FG, 2,2-diFG), and protein synthesis (Figure 4—2-DG, 2-FG, 2,2-diFG). The derivatives we tested were 2-DG, 2-FG, 2,2-diFG, 2-BG, and 2-CG. We also calculated the IC_50_ concentrations of each compound. Our findings, summarized in Table 1, showed that the fluorine-substituted 2-DG derivatives (2-FG, 2,2-diFG) exhibited the highest cytotoxicity, while 2-CG and 2-BG were found to be ineffective (IC_50_ > 20 mM). Interestingly, the activity of 2-FG was similar in both U-251 and U-87 cell lines, whereas 2,2-diFG, was significantly more potent in U-251 cells.

### 3.2. Hypoxia-like Conditions Modulate Fluorinated-2-DG Derivatives Action

Monteiro et al. [60] found that low oxygen levels in tumors increase glucose metabolism in GBM cells, making them more dependent on glycolysis. We hypothesize that GBM cells may be more susceptible to glycolysis inhibition under hypoxic conditions compared to normoxic conditions. To test this, we created hypoxia-like conditions using non-toxic concentrations of the prolyl-4-hydroxylase inhibitor—DMOG [50 μM], which elevates hypoxia-inducible factor (HIF), and rhodamine (Rho) [0.25 μM], an inhibitor of mitochondrial oxidative phosphorylation. We have previously detailed the methods in our publication [61]. It is worth noting that in vitro studies have demonstrated that chemically induced hypoxia-like conditions closely resemble the cellular effects observed in hypoxia chambers. However, the role of immune system activity, which is significant in in vivo studies but not relevant for in vitro experiments, is the primary distinction between the two [62].

**Figure 5:**
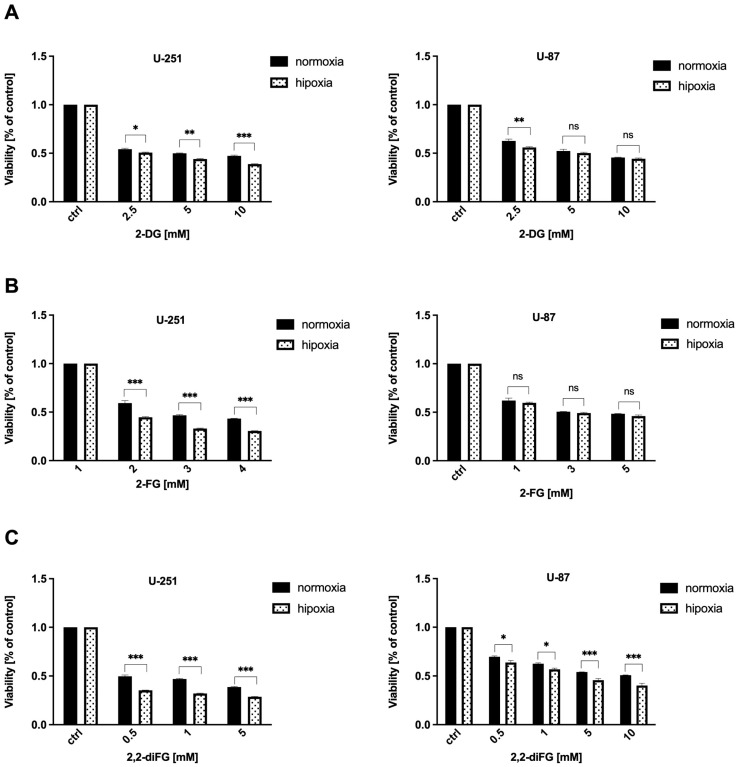
Viability of U-87 and U-251 cells after 72 h treatment with various concentrations of (**A**) 2-DG [0.5–20 mM] and its halogen derivatives: (**B**) 2-FG [1–10 mM], (**C**) 2,2-diFG [0.5–15 mM] in normoxia and hypoxia-like (DMOG + Rho) conditions. Significant differences between the treatment and control means are indicated by * *p* < 0.05, ** *p* < 0.01, *** *p* < 0.001, ns—no statistical significance.

The results depicted in Figure 5 show that hypoxic conditions significantly altered the response of GBM cells to fluorinated 2-DG derivatives, especially 2,2-diFG (Figure 5C). The cytotoxic effect of 2,2-diFG was found to be more potent in both tested cell lines under hypoxia-like conditions. However, in the case of 2-FG, the presence of hypoxia-like changes sensitized only U-251 cells to its cytotoxic action, while the viability of U-87 cells following 2-FG treatment was similar in both normoxic and hypoxic conditions (as indicated in Figure 5B). These findings support the earlier claim that GBM cells are among the most glycolytic tumors [3].

### 3.3. Lactate Levels Decrease in Response to Fluorinated 2-DG Derivatives Treatment

**Figure 6:**
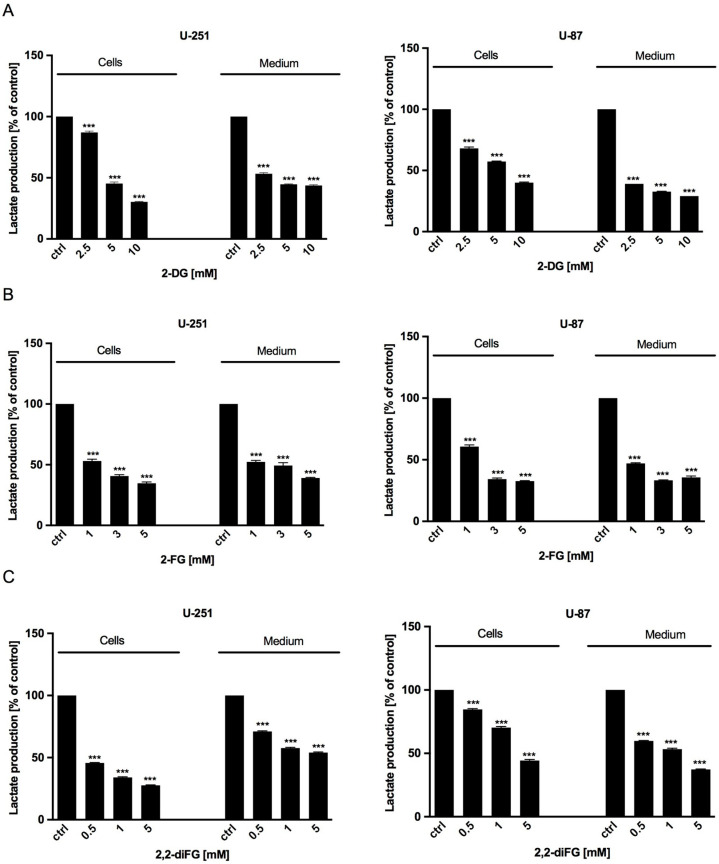
Intracellular (cells) and extracellular (medium) lactate production of U-251 and U-87 cells after 72 h treatment with various concentrations of (**A**) 2-DG [2.5–10 mM] and its halogen derivatives: (**B**) 2-FG [1–5 mM], (**C**) 2,2-diFG [0.5–5 mM]. Significant differences between the treatment and control means are indicated by *** *p* < 0.001.

After exposing the cells to the tested fluorinated 2-DG analogs for 72 h, we measured the synthesis of intracellular and extracellular lactate to evaluate the intensity of glucose utilization through the glycolysis process. Our findings showed that the lactate levels in U-251 and U-87 cells and the culture medium decreased in a dose-dependent manner compared to untreated cells (Figure 6). This decrease corresponded to reduced viability, proliferation, and protein synthesis, as described above.

### 3.4. Autophagy Does Not Mediate Fluorinated 2-DG Derivatives-Induced Cytotoxic Effects

Autophagy is an important process that occurs within cells. It has a dual role in tumor growth, as it can both contribute to cancer cell growth and drug resistance while also acting as a cell death mechanism in response to certain stimuli [63]. Research has shown that inhibition of glucose metabolism and ATP synthesis, as well as glucose deprivation, are major triggers for autophagy through the AMP-activated protein kinase (AMPK) kinase pathway [64]. To investigate the involvement of autophagy, we tested derivatives in the presence of chloroquine (CQ) [10 μM]. CQ is a well-known inhibitor of autophagy that changes the acidic environment of the lysosome, thus preventing the fusion of autophagosomes with lysosomes, which is responsible for blocking further autophagy processing [65].

**Figure 7:**
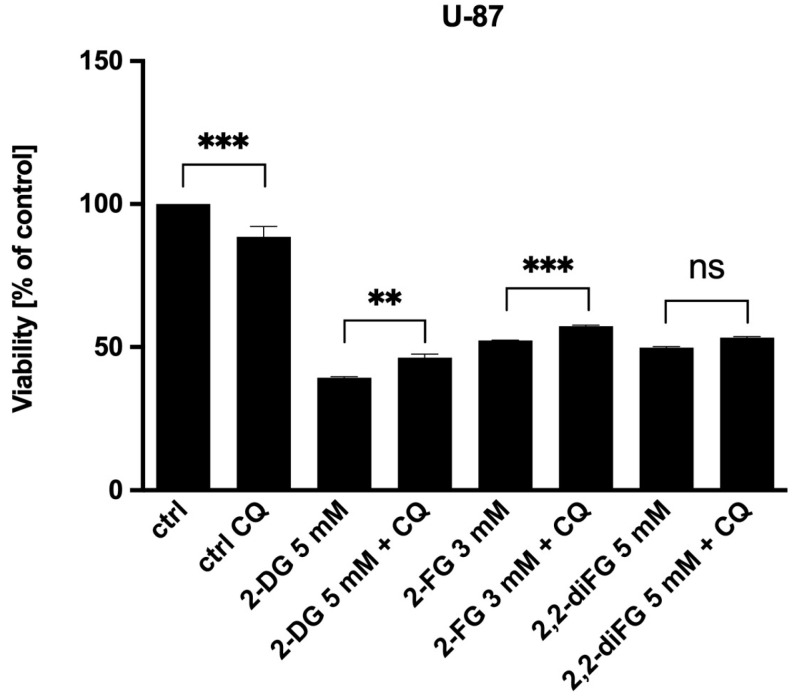
Viability of U-87 cells after 72 h combined treatment of CQ [10 μM] with IC_50_ concentrations of 2-DG [5 mM], 2-FG [3 mM], and 2,2-diFG [5 mM]. Significant differences between the treatment and control means are indicated by ** *p* < 0.01, *** *p* < 0.001, ns—no statistical significance.

We have found that autophagy does not play a significant role in the cytotoxicity of glycolysis inhibitors. Our experiment showed that the addition of CQ only slightly increased the viability of U-87 cells in response to 2-DG and 2-FG (Figure 7). Although a change in cell viability was observed, it did not exceed 10%. Similar observations were made for U-251 cells. As a result, we conclude that autophagy is not the main pathway of cell death induction via fluorinated 2-DG derivatives.

### 3.5. In Vitro Affinity of Halo-2-DG-Derivatives to HKII

**Figure 8:**
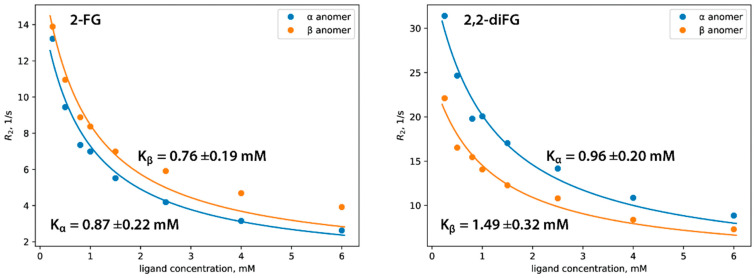
Affinity of halogenated analogs of 2-DG to hexokinase determined by ^19^F NMR relaxation experiment. In each case, the *K_d_* was determined separately for α and β anomers, and the corresponding fitting curves are depicted as blue and orange, respectively.

We used ^19^F NMR spectroscopy, specifically the CPMG pulse sequence, to determine the affinity of halo-2-DG derivatives to HKII and their dissociation constants (*K*_d_). This method measures nuclear-spin transverse relaxation (*R*_2_), which is related to changes in the rotational diffusion (*D*_r_) of the ligand molecule. As the ligand binds to the protein, its apparent *D*_r_ decreases, leading to a change in the *R*_2_ measured in the experiment. By conducting a series of CPMG experiments with varying ligand concentrations (called NMR titration), one can plot *R*_2_ against ligand concentration to determine the *K*_d_ of the protein-ligand complex (Figure 8) (Tables S1–S5). For more information on NMR relaxation experiments related to drug discovery, please refer to other resources [66,67]. To properly determine *K*_d_ for 2-FG and 2,2-diFG, we adapted a commonly used model, which assumes that ligands exist only in one isomeric form. Our modification accounted for the mutarotation of the pyranose ring, leading to the presence of both anomers in the solution. This added complexity necessitated certain simplifications of the model for stable model fitting to the experimental data. A detailed derivation of this adapted model is available in Supplementary Materials (Equations (S1) and (S2)).

Our research shows that both compounds bind to HKII with similar affinities in the micromolar range, as shown in Table 2. The α and β anomers of 2-FG have almost the same binding affinity, while HKII slightly prefers the α anomer of 2,2-diFG. We provide a detailed discussion of these findings and a possible explanation on a structural level in the next paragraph. Compared to other hexokinase inhibitors, such as recently described glucosamine derivatives or benitrobenrazide, our fluorinated 2-DG derivatives show moderate affinity to HKII. Although these compounds have relatively weak binding, our biological results indicate that they can block glycolysis, suggesting that direct binding to hexokinase may not be a primary mode of action.

We aimed to determine the affinity of halogenated 2-DG derivatives (2-CG and 2-BG) using a competitive ^19^F NMR titration experiment. In this method, a fluorinated compound (2-FG) acts as a probe and competes with a second ligand for the binding site. By monitoring changes in the binding equilibrium between the probe and the protein through NMR, we can indirectly determine the *K*_d_ of the investigated ligand. However, it was necessary to adjust the data fitting approach to account for the anomeric equilibria that could not be solved without oversimplification of the studied system. As a result, we provided only a qualitative analysis of NMR data to compare binding affinities within the set of investigated ligands. The relaxation rates of 2-FG gradually decreased with increasing concentrations of 2-DG, indicating the capability of this compound to interact with the same binding site as the probe. However, increasing concentrations of either 2-CG or 2-BG did not significantly alter the relaxation rates of 2-FG, suggesting that both compounds are relatively weak competitive binders. The obtained relaxation rates of tested compounds are presented in Supplementary Materials (Tables S1–S4).

### 3.6. Molecular Docking of Halo-2-DG Derivatives to HKII and Analysis of Binding

We performed molecular docking of fluorinated analogs of 2-DG to gain a better understanding of the mechanism of HKII inhibition. We also included 2-BG and 2-CG for comparative studies.

HKII can bind both glucose and its phosphorylated derivative, glucose-6-phosphate (G-6P), with G-6P acting as a product of HKII enzymatic activity and its allosteric regulator inhibiting the activity of this enzyme [68]. Glucose binding induces a conformational change that blocks ATP hydrolysis, which is reported in all crystallographic structures of hexokinases I-III. To explore the differences in biological activity among the studied compounds, we utilized existing HKII structures complexed with glucose and G-6P (PDB entry NZT2). We applied the DockThor web server, which is optimized to handle highly flexible ligands, including polypeptides [56,57]. We validated our docking approach by successfully docking glucose in two modes: ‘blind docking’, without a predefined binding site, and ‘targeted docking’, focusing on the glucose binding cavity and its vicinity. The computed binding energies are summarized in Table S5. In the blind docking mode, DockThor could not find the expected binding cavity, and all ligands, including glucose, were placed in the binding cavity for G-6P. However, in targeted mode, both 2-DG and its analogs were correctly placed. The binding and total energies for the highest-ranked showed minimal differences in the affinities of the ligands for HKII. This is consistent with the known conformational flexibility of HKI and HKII, which can accommodate various glucose derivatives. Additionally, most docking scoring functions, due to computational constraints, may not fully account for subtle intermolecular interactions, such as halogen bonding or dispersive forces [29]. Furthermore, machine learning-based scoring functions often require more extensive datasets for improved accuracy [69].

**Figure 9:**
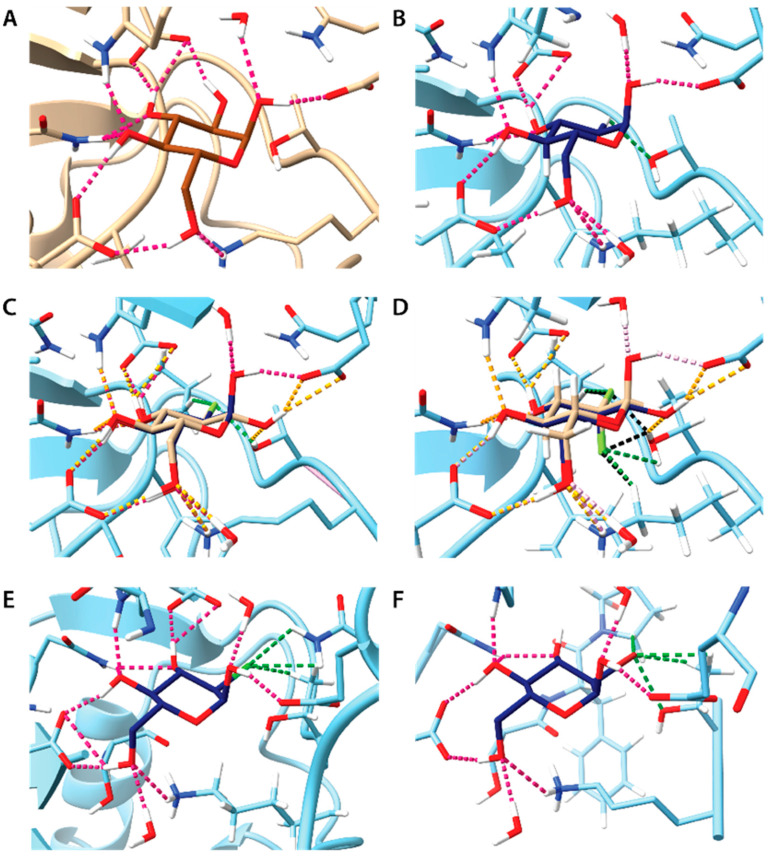
Molecular docking of 2-DG and its derivatives to crystal structure HKII (PDB entry 2NTZ). In each panel, selected hydrogen atoms and amino acid residues of HKII were omitted for clarity, making binding site visible. Typical H···O hydrogen bonds are depicted as pink dotted lines. H-bonds with less stringed geometrical constraints and close contacts involving position 2 in the pyranose ring are depicted as green dotted lines. If not mentioned otherwise only the α anomers are shown. Panels A-F show the docking of following compounds: (**A**) Glc (experimental data from literature, PDB entry 2NTZ); (**B**) 2-DG; (**C**) 2-FG, superposition of both anomers, H···O hydrogen bonds formed by the β anomer are depicted as orange dotted lines, H···F hydrogen bonds are depicted as green dotted lines; (**D**) 2,2-diFG, superposition of both anomers, H···O hydrogen coloring scheme is as in the panel C, H···F hydrogen bonds are depicted as dotted lines colored green and black for anomers α and β, respectively; (**E**) 2-CG; (**F**) 2-BG.

A closer inspection of ligand binding poses indicates subtle differences in intermolecular interactions with HKII (Figure 9). The absence of a 2-OH group in 2-DG limits the formation of a strong hydrogen bond (H-bond) with E708, allowing only a weak C-H···O interaction with T620. In contrast, fluorine substitution in 2-FG results in a better fit to the binding cavity and more attractive interactions. The H-bonding at the C-2 position involves C-H···F and O-H···F bonds with P605 and T620, respectively. An almost identical H-bond pattern is observed for both α and β anomers of 2-FG, potentially explaining the small differences in their dissociation constants (*K*_d_) observed in the NMR experiment. In the case of 2,2-diFG, the double fluorine substitution leads to a slightly altered H-bond pattern at the C-2 position, aligning with the NMR results showing some difference in *K_d_* between its α and β anomers. The presence of chlorine in 2-CG, with its slightly larger atomic size, does not significantly alter the geometry of H-bonds outside the C-2 position. This suggests that the local effects at the C-2 position are likely responsible for the observed lack of binding in NMR studies. Conversely, in the case of 2-BG, the larger bromine atom necessitates a different ligand orientation, partially disrupting the H-bond network, which also may reduce its affinity.

To further analyze protein-ligand interactions, we applied Hirshfeld surface (HS) analysis, a technique widely employed in crystallography to visualize intermolecular interactions [70] (Table S6). The HS delineates a boundary around a molecule in a crystal where the electron density contributions from the atoms of the molecule itself (promolecule) and those from neighboring molecules in the lattice (procrystal) are equal. In the case of protein-ligand interactions, it is assumed that the ligand is the promolecule and the binding cavity represents the crystal environment. HS analysis quantifies intermolecular interactions in crystal structures, showing the contribution of each type of interatomic contact. In HS analysis, two critical parameters are used: *d*_i_ and *d*_e_, which represent the nearest distances from any point on the HS to an atom nucleus inside (*d*_i_) or outside (*d*_e_) the surface, respectively. These distances are instrumental in visualizing intermolecular contacts when applied to so-called “fingerprint plots”, showing the distribution of the interatomic contacts in the crystal structure as a function of both parameters.

**Figure 10:**
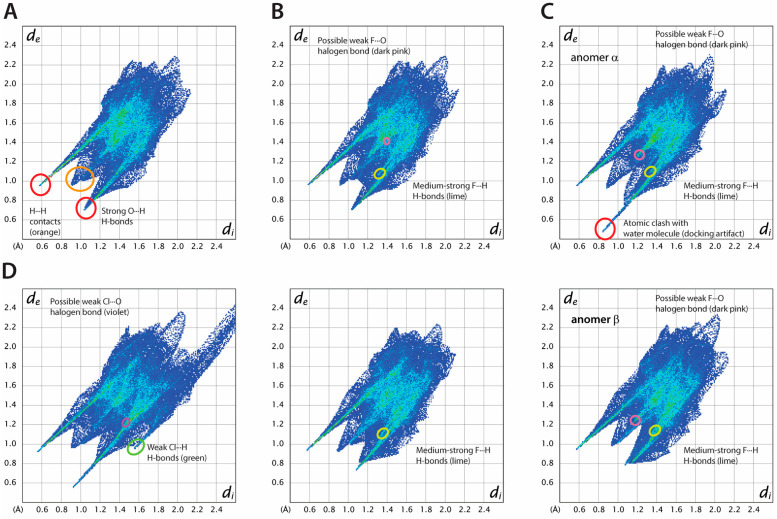
Hirshfeld surface analysis of ligand-protein interactions for selected compounds (**A**) 2-DG (red—strong O···H H bonds, orange—H···H contacts), (**B**) 2-FG, anomers α and β are shown on top and bottom, respectively (dark pink—possible weak F···O halogen bond, lime—medium strong F···H H-bonds, (**C**) 2,2′-diFG, anomers α and β are shown at top and bottom, respectively (red—atomic clash with water molecules (docking artifact), dark pink—possible weak F···O halogen bond), (**D**) 2-CG (violet—possible weak Cl···O halogen bond, green—weak Cl···H H-bonds, lime—medium strong F···H H-bonds, dark pink—possible weak F···O halogen bond).

For each ligand, the most prominent feature of fingerprint plots are diagonally symmetric, sharp “spikes”, which are the presence of strong O-H···O and N-H···O H-bonds between the ligand and amino acid residues (Figure 10).

Minor changes are observed in the fingerprint plots when fluorine atoms are introduced, suggesting that glucose, 2-DG, and its fluorinated analogs bind similarly. This is in line with the general belief that fluorine is a useful bioisostere for hydrogen and the OH group [71,72]. The percentage of H···F contacts increases with the number of fluorine atoms, while the opposite trend is observed for H···H contacts. On the other hand, the number of O···H contacts is only slightly reduced, as the pattern of O(N)-H···O bonds in each compound remains the same (see Table S6 for details). While there is some accumulation of specific F···O contacts on the HS of 2-FG and 2,2-diFG, it is doubtful that there is any halogen bonding with the fluorine atom. Only a few reports suggest the existence of such interactions, typically occurring when fluorine is close to strong electron-withdrawing groups [73].

The presence of bulkier substituents, such as in the case of 2-CG and 2-BG, causes significant changes in the fingerprints, with more scattered interatomic contacts and less dense binding. These compounds have weak C-H···X (where X is a halogen) hydrogen bonds, which might partially offset the destabilizing impact of the halogen. The F < Cl < Br series shows a monotonic rise in H···X contacts, primarily due to the increasing radius of the substituent. The halogen atoms in both 2-BG and 2-CG participate in specific interatomic contacts with oxygen atoms, indicating the presence of halogen bonds (XBs), with nearby oxygen atoms acting as acceptors. The estimated values of *θ*_1_ valence angle (R-X_1_···X_2_) and X_1_···X_2_ distances for these interactions (~90–100°, 2.6–2.8 Å, respectively) align with the characteristics of type II XB. In several systems, there is a subtle interplay between hydrogen bonds and XBs, which can be cooperative [74] or competitive [75]. According to our HS analysis, halogen atoms in the studied protein-ligand complexes can be involved in both types of interactions. However, determining their precise nature requires further experimental or computational studies.

The comparison of fingerprint plots for glucose, both in its crystalline state (Figure S2) and when bound to hexokinase, shows that they have very similar interatomic contacts and packing density. This similarity is reflected in their fingerprint plots. In both cases, the primary contributions to intermolecular interactions are strong H-bonds, with a consistent pattern despite the differences in chemical environments.

There are certain similarities in the structures of HKII complexed with different known inhibitors, such as 2–6-substituted glucosamines. These inhibitors occupy the glucose binding cavity, and their pyranose rings establish hydrogen bond networks similar to glucose. However, their elongated substituents at the C-2 position partially intrude into the 6-GP binding site, leading to strong inhibitory potential. This observation highlights the flexibility of HKII, which allows for various glucose analogs to bind, including analogs of 2-DG and substituted glucosamine derivatives. While there is no experimental structural research on halogenated derivatives of 2-DG, a computational study on their binding to HKI, which is structurally very similar to HKII, has been conducted. The study used different methods from ours (the authors applied geometry optimization using a customized force field for manually created protein-ligand models), but their findings and overall conclusions were similar, showing that an increase in halogen atom size reduces the ligand’s affinity. As a result, 2-FG, with its smaller substituent, fits well in the binding pocket, potentially explaining its enhanced inhibitory potential when compared to 2-DG [48,65].

### 3.7. 6-Phosphates of 2-DG and Its Halo-Derivatives Differentially Modulate HK Activity

**Figure 11:**
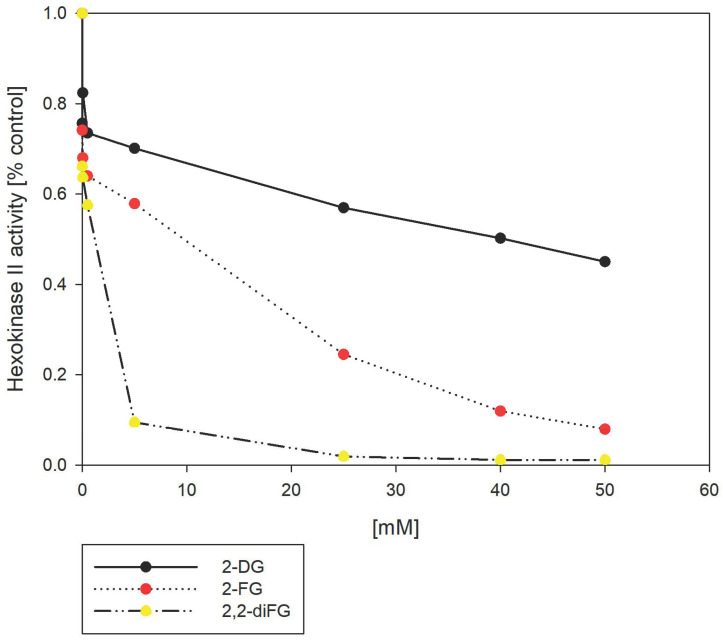
Inhibition of HKII by 2-DG and its fluorinated-derivatives (2-FG, 2,2-diFG). The HKII activity is normalized to 1.0 in the absence of any inhibitors.

In the cellular environment, 2-DG is transformed into 2-deoxy-6-phosphate (2-DG-6P) by HKII, which then accumulates inside the cell [23]. We hypothesized that the ability of various 2-DG derivatives to inhibit glycolysis could be linked to their interactions with HKII. To test this hypothesis, we used a hexokinase II inhibitor screening assay that measures the capacity of the tested compounds to inhibit the recombinant HKII enzyme. The assay detects the oxidation of glucose-6-phosphate (G-6P), produced by HKII, via glucose-6-phosphate dehydrogenase. This enzyme cannot process other pyranose-6-phosphates, including 2-DG-6P. As shown in Figure 11, our results showed that fluorinated 2-DG derivatives were more potent HKII inhibitors than 2-DG itself. Notably, 2,2-diFG emerged as the most potent inhibitor under in vitro conditions. However, the significant inhibitory potential observed in the enzymatic test, as well as cytotoxicity, are mostly caused by products of HKII activity, namely 6-phosphates of the tested compounds. In physiological conditions, G-6P is an allosteric inhibitor of HKII, and a similar inhibitory mechanism is expected from 6-phosphates of 2-DG. Previous studies have shown that 2-DG-6P is a relatively weak inhibitor of HKII with K_I_ of 0.24 mM, which may explain its low cytotoxicity and inhibitory effect in vitro [76,77]. To understand the discrepancies in the results of the mentioned enzymatic test, we performed molecular docking of their 6-phosphates, using the same methods as in the case of 2-DG and its derivatives. However, these results are inconclusive (Figure S3), as the computed binding pose of 2-DG-6P is different from the known pose of 6-GP in the crystal structures of HKI and HKII. It is rather unexpected that a single substitution of the OH group by a hydrogen atom should have a profound impact on the ligand pose. Hence, these findings imply that docking of other pyranose-6-phosphates may be inaccurate and should be interpreted with caution.

## 4. Discussion

### 4.1. Effects of 2-DG and Its Halogenated Derivatives on Cellular Metabolism

It has been nearly 70 years since 2-DG was first discovered as a glycolysis inhibitor and potential antiviral and anticancer agent [78,79]. Because of its limited systemic toxicity, the use of 2-DG in treatments has been explored over the years. Recently, a clinical trial was conducted in India to investigate the effectiveness of 2-DG as an experimental COVID-19 therapy. The study found that using a dosage of 90 mg/kg/day of 2-DG in addition to the Standard of Care (SOC) was more beneficial in treating moderate to severe COVID-19 than SOC alone [80]. However, previous clinical trials in cancer patients did not show significant efficacy of 2-DG administration [11,17].

Chemical modification of 2-DG is being explored to improve its pharmacokinetics and clinical efficacy. For example, acetylated and di-acetylated 2-DG derivatives have been synthesized as potential antimetabolites [81]. Fluorination of 2-DG is also commonly performed to prepare radiolabeled 2-FG for Positron Emission Tomography (PET) imaging. Among halogenated 2-DG derivatives, only 2-FG has been reported to have a potent cytotoxic effect. A recent study showed that 2-FG was able to inhibit glucose uptake in xenograft tumors and sensitized HeLa cancer cells to doxorubicin in both hypoxia and normoxia, whereas 2-DG was active only under normoxia [82]. In a pancreatic model, fluorinated 2-DG derivatives exhibited superior ability to inhibit glycolysis in vitro compared to 2-DG, especially in hypoxia conditions [83]. Moreover, 2-FG-mediated glycolysis downregulation appeared to be highly efficient in inhibiting the intraerythrocytic stage of the human malaria parasite since *Plasmodium falciparum* growth and multiplication relies on glycolysis for ATP generation [49]. The biological activity of other halogenated 2-DG derivatives has been investigated primarily by Priebe’s group [48]. They reported minimal anticancer activity of 2-CG and 2-BG, while 2-FG was found to be a potent glycolysis inhibitor. The authors suggested that the distinct ability of halogenated 2-DG derivatives to inhibit HKII activity may be the reason for this difference [48]. Our computational and biochemical investigation also supports this conclusion, which will be further discussed in the following paragraphs.

### 4.2. Binding Affinities of 2-DG and Its Halogenated Derivatives and Their Impact on Biological Activity

NMR experiments have shown that 2-DG and its halogenated derivatives have only moderate binding affinity to HKII in vitro, with *K*_d_ values in the micromolar range. While there are no reports directly comparable to our findings for glucose or its close structural analogs, the Michaelis constants (*K*_M_) for glucose, as reported in the BRENDA database, are below 0.5 mM on average, which falls within a similar molar range as in our binding experiments [84]. It is important to note that *K*_M_ and *K*_d_ are different parameters: *K*_M_ is a kinetic constant and describes the rate of an enzymatic reaction, while *K*_d_ is a thermodynamic parameter that indicates the stability of the ligand-target complex. However, both parameters can have comparable values in practice, assuming that the substrate is in instantaneous chemical equilibrium with the substrate-enzyme complex [85]. In a previous study, the *K*_M_ values for glucose, 2-DG, and 2-FG in HKII from *Rattus norvegicus* were 0.13, 0.61, and 0.17 mM, respectively [86]. This suggests that glucose and 2-FG have higher affinities for HKII compared to 2-DG. Despite differences in the reported values of *K*_M_ for 2-DG in publications, the constant for 2-DG is consistently 2–8 times higher than for glucose in the context of a given experiment. Our findings indicate that 2-DG, 2-FG, and 2,2-diFG have moderate affinity to HKII, comparable to that of glucose, while 2-CG and 2-BG are weaker binders. Molecular docking supports these results, showing variations in intermolecular interactions, primarily influenced by the size and polarity of the substituent at the C-2 position. The series F > Cl > Br suggests an inverse relationship between substituent size and the ability to form strong H-bonds, with larger atoms favoring the formation of halogen bonds, as previously proposed [48]. These interactions, particularly involving bromine, can be as strong as H-bonds and play a significant role in drug-protein interactions, which have been scrutinized in recent articles [47,87,88]. However, in the case of 2-DG derivatives, their contribution to overall binding seems to be limited.

Despite its high inhibitory potential, 2-BG 6-phosphate has only a weak toxic effect on glioblastoma cells. This may be because bromo-modified sugars are less chemically stable, leading to rapid degradation and the lower cytoplasmic levels of 2-BG and its 6-phosphates. However, this hypothesis requires further experimental verification.

Based on our observations, 2-CG 6-phosphate seems to be a weaker inhibitor of hexokinase and is significantly less potent than other halogenated sugars. Our findings suggest that the overall effectiveness of these compounds as potential therapeutic agents depends on a nuanced balance between chemical stability, cellular accumulation, and glycolysis inhibition.

### 4.3. 6-Phosphates of Glucose and Its Analogues Have Different Binding Properties

Assuming that the 6-phosphates of the compounds being investigated bind to HKII like the natural ligand, it is proposed that changes in affinity are mainly driven by varying intermolecular interactions near the C-2 position. The substitution of the OH group by hydrogen in 2-DG-6P disrupts two hydrogen bonds, thus leading to a decrease in its affinity for HKII. Understanding the effects of other substituents is more challenging. In the case of 2-FG, the improved affinity may be attributed to the fluorine atom’s ability to form relatively strong H···F hydrogen bonds. Similarly, the CF_2_ group in 2,2-diFG might create a network of similar interactions, thereby enhancing the stability of the protein-ligand complex. The contrast between the inhibitory properties of 2-CG and the more potent 2-BG is notable. It is speculated that the introduction of a bulkier chlorine atom may disrupt binding to the allosteric center, whereas the larger bromine atom in 2-BG could result in an altered mode of binding forced by steric hindrance. This may obstruct access to the allosteric center or even lead to significant rearrangements of the nearby amino acid residues. However, these hypotheses need to be verified by further experimental structural studies.

The crystal structures of HKI and HKII show that both proteins have similar structures and binding modes for both glucose and G-6P in their catalytic C-terminal domains [89]. A preliminary structural analysis of HKI interactions with 2-DG-6P and mannose 6-phosphate suggests that both compounds bind similarly to G-6P as observed in Figure S4, panels C and D. However, mannose 6-phosphate is known to be a more effective hexokinase inhibitor [77,90]. This indicates that even minor structural differences, such as the position of an OH group in a pyranose-6-phosphate, can significantly influence its allosteric properties. Our results support this, demonstrating that chemical modifications at the C-2 position substantially alter the biochemical properties of pyranose-6-phosphates, which in turn lead to different outcomes in cellular studies.

Existing HKII inhibitors face several significant limitations that impede their clinical application. A primary challenge is the lack of specificity, which often results in off-target effects and toxicity in normal cells that also rely on glycolysis for energy, thereby limiting the therapeutic window. Inhibitors that target the glucose or G-6P-binding sites often contain polar fragments that can hinder their permeability across biological barriers, resulting in poor bioavailability and rapid clearance from the body. Some inhibitors, although effective in vitro, do not exhibit sufficient potency in vivo or fail to disrupt HKII binding to its mitochondrial binding partner, voltage-dependent anion-selective channel 1 (VDAC1), which is critical for its anti-apoptotic function. The crystal structure of the HKII-VDAC1 complex is not yet available, complicating the design of inhibitors that can effectively disrupt this interaction [91]. As a result, inhibitors designed through virtual screening or structure-based design may not fully exploit key molecular interactions. Moreover, delivery challenges persist, as effectively targeting tumors while minimizing systemic exposure remains difficult. Lastly, the limited number of clinical trials and insufficient data on safety and efficacy in humans restrict the translation of some promising preclinical findings into viable treatment options. Addressing these issues is crucial for the successful development of HKII inhibitors as effective anticancer therapies.

## 5. Perspectives

Cancer cells, particularly in aggressive tumors like GBM, heavily rely on glycolysis (Warburg effect). This heightened dependence on glycolysis renders glycolysis inhibitors potentially more selective for cancer cells compared to normal cells, presenting an attractive therapeutic strategy.

Our results indicate that fluorinated 2-DG derivatives (2-FG, 2,2-diFG) exhibit greater potency in inhibiting glycolysis than 2-DG. This increased efficacy is primarily due to better inhibiting HKII and other downstream glycolytic enzymes. Since glycolysis serves as a major energy source for GBM, these analogs may more effectively disrupt cancer metabolism. The superior inhibition of HKII by halogenated analogs, particularly through their interaction with the 6-phosphate form, advances our understanding of refining glycolytic inhibitors to target specific enzymes more efficiently. Furthermore, GBM tumors are often highly hypoxic, which strengthens their reliance on glycolysis. The fluorinated analogs demonstrate enhanced cytotoxic effects under hypoxic conditions, making them particularly well-suited to targeting GBM in its unique tumor microenvironment. Moreover, the glycolysis inhibitors may exhibit synergistic effects when combined with other therapies such as radiation or immune checkpoint inhibitors. By reducing the energy supply of cancer cells, these inhibitors could render them more vulnerable to additional stressors [92]. While our study focuses on GBM, the findings may be applicable for other cancers that heavily depend on glycolysis, including pancreatic, colorectal, or lung cancers. These promising observations pave the way for designing more potent analogs, potentially broadening the scope of glycolysis-targeted cancer therapies. However, it is important to note that our studies represent only the initial steps in developing new anticancer drug candidates. While our fluorinated 2-DG derivatives exhibit potent cytotoxic effects in vitro, their long-term efficacy and safety in vivo have yet to be fully established. Future studies should include animal models to assess these compounds’ therapeutic potential, biodistribution, and potential toxicity over extended periods. This will allow us to determine whether the improved efficacy observed in cell culture translates into meaningful benefits in living organisms and identify any potential adverse effects. Furthermore, although our study provides insights into the interactions between the fluorinated analogs and hexokinase II, a deeper mechanistic understanding of how these compounds affect other components of the glycolytic pathway and overall cellular metabolism is needed. Additional research should focus on elucidating the broader metabolic impact of these analogs, including their effects on related metabolic pathways and potential off-target effects. We should also consider the potential mechanisms that GBM cells might develop in response to fluorinated analog treatment, which will be crucial for optimizing animal studies and preventing resistance. Future studies should also explore combination therapies or adjuvant treatments that could mitigate resistance and enhance the effectiveness of these analogs. Finally, although our fluorinated analogs show improved pharmacokinetic properties compared to 2-DG, further research is required to refine their formulation and delivery methods. These studies should focus on optimizing bioavailability, stability, and targeted delivery to ensure that these compounds can achieve effective concentrations at the tumor site while minimizing systemic toxicity.

From a broader perspective, our study can be viewed as part of a larger trend in chemical biology focusing on further development of fluorinated carbohydrates in fields of both drug discovery and biological applications. These compounds are poised to play a bigger role in drug discovery, particularly in the design of glycomimetics and enzyme inhibitors. Fluorination can improve the pharmacokinetic properties of carbohydrate-based drugs, such as their stability, bioavailability, and binding affinity to targets like glycosidases and glycosyltransferases. Furthermore, there is significant potential for using fluorinated carbohydrates in biological studies, such as probing glycan-protein interactions, studying membrane transport mechanisms, and exploring carbohydrate-related metabolic pathways. These derivatives are also expected to contribute more to imaging techniques, such as PET scans, where fluorinated glucose analogs are already in use.

## Figures and Tables

**Table 1:** IC_50_ concentrations of 2-DG and its halogen derivatives.

Tested Derivative	IC_50_ Concentration [mM]	
	U-251 Cell Line	U-87 Cell Line
2-DG	5	5
2-FG	2.5	3
2,2-diFG	0.5	5
2-BG	not specified, >20	not specified, >20
2-CG	not specified, >20	not specified, >20

**Table 2:** Dissociation constants (*K*_d_) and relaxation rates (*R*_bound_) of the bound form for fluorinated ligands.

Compound	*K*_α_ [mol m^−3^]	*K*_β_ [mol m^−3^]	*R*_bound_ [s^−3^]
2,2-diFG	0.96 ± 0.20	1.49 ± 0.32	3200 ± 440
2-FG	0.87 ± 0.22	0.76 ± 0.19	1310 ± 190

## Data Availability

The data presented in this study are available on request from the corresponding authors.

## Supplementary Materials

The following are available online at https://www.mdpi.com/article/10.3390/biomedicines12102240/s1, Equation (S1): Determination of dissociation constant of fluorinated ligands; Equation (S2): Determination of dissociation constant of non-fluorinated ligands; Table S1: Relaxation rates for 2,2-diFG; Table S2: Relaxation rates for 2-FG; Table S3: Relaxation rates for 2-DG; Table S4: Relaxation rates for 2-CG; Table S5: Docking results from DockThor server; Table S6: Percentage contributions to the Hirshfeld surface area of each type of intermolecular contact for 2-DG and its analogs bound to HKII; Figure S1: Viability of U-251 cells after 72 h treatment with various concentrations of halogen 2-DG derivatives; Figure S2: Fingerprint plots for selected protein-ligand complexes relevant to the study; Figure S3: Results of molecular docking of 6-phosphates of glucose and its derivatives to HKII; Figure S4: Testing of DockThor method.
