# Supplementary material for: Potent Biological Activity of Fluorinated Derivatives of 2-Deoxy-d-Glucose in a Glioblastoma Model
Source: Biomedicines. 2024 Oct 1;12(10):2240. doi: 10.3390/biomedicines12102240 (PMC11504489; doi:10.3390/biomedicines12102240)
Supplement: Supplementary file 1 [file biomedicines-12-02240-s001.zip › biomedicines-3219524-supplementary.pdf]

Supplementary materials:

Equation (S1). Determination of dissociation constant of fluorinated ligands

For the system consisting of a protein and a ligand existing as a mixture of two forms being in a dynamic equilibrium (for instance a mixture of two anomers), the whole process can be summarized as follows:

$$PL_\alpha + L_\alpha \underset{K_\alpha}{\leftrightarrows} P + L_\alpha + L_\beta \underset{K_\beta}{\rightleftarrows} PL_\beta + L_\beta$$

where: P – protein, L_α / L_β– ligand in α / β anomeric form, PL_α / PL_β – protein bound with α / β form of the ligand, K_α / K_β – dissociation constant describing interaction of the protein with α / β form of the ligand.

From the above scheme we get the following dependencies:

$$K_\alpha = \frac{[P]\cdot[L_\alpha]}{[PL_\alpha]} \qquad (1)$$

$$K_\beta = \frac{[P]\cdot[L_\beta]}{[PL_\beta]} \qquad (2)$$

where the brackets denote molar concentrations of the components.

Assuming that total concentrations of protein and ligand are known ($[P_0]$) and $[L_0]$, respectively), we may obtain:

$$[L_\alpha] + [PL_\alpha] + [L_\beta] + [PL_\beta] = [L_0] \qquad (3)$$

$$[PL_\alpha] + [PL_\beta] + [P] = [P_0] \qquad (4)$$

Assuming constant ratio of anomers concentrations, we get the last equation:

$$s = \frac{[L_\alpha]}{[L_\beta]} \qquad (5)$$

Solving the above set of equations yields:

$$[PL_\alpha] = \frac{-b-\sqrt{\Delta}}{2\cdot a} \qquad (6)$$

$$[PL_\beta] = \frac{K_\alpha\cdot(-b-\sqrt{\Delta})}{2\cdot a\cdot s\cdot K_\beta} \qquad (7)$$

$$[L_\alpha] = s\cdot\frac{[L_0]+\frac{b+\sqrt{\Delta}}{2\cdot a}\cdot\left(1+\frac{K_\alpha}{s\cdot K_\beta}\right)}{1+s} \qquad (8)$$

$$[L_\beta] = \frac{[L_0]+\frac{b+\sqrt{\Delta}}{2\cdot a}\cdot\left(1+\frac{K_\alpha}{s\cdot K_\beta}\right)}{1+s} \qquad (9)$$

where:

$$a = s\cdot K_\beta + 2\cdot K_\alpha + \frac{K_\alpha^2}{s\cdot K_\beta} \qquad (10)$$

$$b = -\left([L_0]\cdot s\cdot K_\beta + [P_0]\cdot s\cdot K_\beta + K_\alpha\cdot[P_0] + K_\alpha\cdot[L_0] + K_\alpha\cdot K_\beta + K_\alpha\cdot K_\beta\cdot s\right) \qquad (11)$$

$$c = [P_0]\cdot[L_0]\cdot s\cdot K_\beta \qquad (12)$$

$$\Delta = b^2 - 4\cdot a\cdot c \qquad (13)$$

For any of the ligand nuclei, the observed transverse relaxation rate can be expressed as:

$$R^{\alpha}_{obs} = R^{\alpha}_{free} \cdot \frac{[L_\alpha]}{[L_\alpha]+[PL]_\alpha} + R^{\alpha}_{bound} \cdot \frac{[PL_\alpha]}{[L_\alpha]+[PL_\alpha]} \qquad (14)$$

(for a nucleus of α anomer) and

$$R^{\beta}_{obs} = R^{\beta}_{free} \cdot \frac{[L_\beta]}{[L_\beta]+[PL_\beta]} + R^{\beta}_{bound} \cdot \frac{[PL_\beta]}{[L_\beta]+[PL_\beta]} \qquad (15)$$

(for a nucleus of β anomer),

where R^{α}_{free} / R^{β}_{free} are transverse relaxation rates of the free forms of α / β anomer, and R^{α}_{bound} / R^{β}_{bound} are transverse relaxation rates of the bound forms of α / β anomer.

Substituting in eq. (14) and (15) the concentrations by formulas (6)-(9) leads to expressions in which the observed relaxation rates depend on: $[P_0]$, $[L_0]$ (which are controlled during the experiment), s, R^{α}_{free}, R^{β}_{free} (which can be determined from NMR experiments) and K_α, K_β, R^{α}_{bound}, R^{β}_{bound} (which are unknown). When the R^{α}_{obs} and R^{β}_{obs} are measured several times for different $[L_0]$ concentrations, it is possible to fit the obtained dependence of $R^{\alpha}_{free}([L_0])$ and $R^{\beta}_{free}([L_0])$ to equations (14) and (15) (with concentrations expressed as in (6)-(9)), respectively. The first fit allows to determine K_α, K_β and R^{α}_{bound}, while the second fit yields K_α, K_β and R^{β}_{bound}. Furthermore, one may expect to obtain consistent results for K_α and K_β from both fits.

Unfortunately, the application of the above procedure did not yield the expected results. We applied two Python packages for non-linear fitting (*symfit* and *scipy.optimize*), and in both cases the results were unstable and prone to falling into local minima. As a result, we decided to introduce a simplification of the equilibria, assuming that $R^{\alpha}_{bound} = R^{\beta}_{bound}$. This is reasonable assumption, as the relaxation rate of a ligand in the protein complex is determined primarily by the relaxation of the protein itself. After this modification the fitting provided stable results (consistent also with brute-force fitting approach), which are reported in the main text of the article.

Equation (S2). Determination of dissociation constant of non-fluorinated ligands

In the case of ligands that do not contain fluorine atoms which can be directly observed by NMR, the binding event can be monitored indirectly, using another fluorine-containing ligand (L_F) of the same protein as a molecular probe. In our case, both fluorinated and non-fluorinated ligand exist as a mixture of two anomeric forms, so the scheme of possible binding events can be depicted as follows:

$$PL_{F\beta} + L_{F\alpha} + L_\alpha + L_\beta$$

$$\uparrow\downarrow\ K_{F\beta}$$

$$PL_{F\alpha} + L_{F\beta} + L_\alpha + L_\beta \underset{K_{F\alpha}}{\leftrightarrows} P + L_{F\alpha} + L_{F\beta} + L_\alpha + L_\beta \underset{K_\alpha}{\rightleftarrows} PL_\alpha + L_{F\alpha} + L_{F\beta} + L_\beta$$

$$K_\beta \uparrow\downarrow$$

$$PL_\beta + L_{F\alpha} + L_{F\beta} + L_\alpha$$

where the notation is similar to the previously discussed case (with only fluorinated ligand), but now labels with lower index F denote quantities related to fluorinated ligand and those without F lower index are quantities related to non-fluorinated ligand.

We can provide equations in analogy to eq. (1)-(5) but due to the presence of two different ligands, a proper description of the equilibria requires nine of such equations. The level of complexity for a system of non-linear equations increases significantly with the number of equations and finding a stable solution may be a non-trivial task. In our case we were unable to solve this system of non-linear equations, despite using computer algebra systems like *Mathematica* and *SageMath*, or the Python simulation framework *SimPy*. Therefore, in this case the results can be described only in a qualitative manner. Upon addition of the L to the solution (containing the protein and the L_F) we observed the decrease of the relaxation rate of the L_F, indicating that the L_F is increasingly removed from the complex and hence, there are certain interactions between the protein and the L_F. Nonetheless, this finding cannot confirm whether dissociation constant of the PL complex is smaller than that of PL_F complex. On the other hand, a larger effect on FL relaxation rate indicates stronger interaction between the protein and L. In a situation where several non-fluorinated ligands are being investigated, they can be ranked according to the strength of the interaction, even if the dissociation constants for the compounds cannot be determined.

Table S1. Relaxation rates for 2,2-diFG.

	α anomer	β anomer
2,2-diFG [mM]	R_2 $[\frac{1}{s}]$	R_2 $[\frac{1}{s}]$
0.25	31.4±1.6	22.1±1.4
0.5	24.65±0.91	16.52±0.60
0.8	19.79±0.47	15.45±0.60
1	20.06±0.79	14.06±0.51
1.5	17.03±0.58	12.27±048
2.5	14.16±0.47	10.80±0.47
4	10.86±0.25	8.38±0.33
6	8.85±0.28	7.30±0.26
2 (no protein added)	2.915±0.073	3.38±0.14

Table S2. Relaxation rates for 2-FG

	α anomer	β anomer
2-FG [mM]	R_2 $[\frac{1}{s}]$	R_2 $[\frac{1}{s}]$
0.25	13.22±0.69	13.89±0.74
0.5	9.44±0.38	10.95±0.42
0.8	7.35±0.12	8.88±0.23
1	6.99±0.20	8.37±0.22
1.5	5.517±0.089	6.99±0.17
2.5	4.189±0.074	5.91±0.13
4	3.153±0.038	4.688±0.067
6	2.627±0.043	3.921±0.050
2 (no protein added)	0.599±0.028	0.806±0.016

Table S3. Relaxation rates for 2-DG

	2-FG α anomer	2-FG β anomer
2-DG [mM]	R_2 [$\frac{1}{s}$]	R_2 [$\frac{1}{s}$]
0.25	3.809±0.072	5.280±0.061
0.5	3.486±0.029	4.898±0.072
1	3.210±0.037	4.368±0.067
1.5	3.078±0.061	4.468±0.070
2.5	2.863±0.046	4.125±0.061
4	2.556±0.042	3.842±0.065
6	2.463±0.014	3.440±0.068
8	2.190±0.021	3.195±0.057
10	2.094±0.018	3.023±0.035

Table S4. Relaxation rates for 2-CG

	2-FG α anomer	2-FG β anomer
2-CG [mM]	R_2 [$\frac{1}{s}$]	R_2 [$\frac{1}{s}$]
0.25	3.740±0.042	5.302±0.055
0.5	3.783±0.047	5.722±0.084
1	3.824±0.039	5.501±0.088
1.5	3.655±0.040	5.35±0.11
2.5	3.713±0.057	5.285±0.078
4	3.604±0.043	5.091±0.090
6	3.450±0.055	4.901±0.066
8	3.358±0.064	4.957±0.058
10	3.397±0.034	4.808±0.078

Table S5. Docking results from DockThor server. Type refers to the docking mode, "targeted" for the predetermined localization of the binding site and "blind" for the blinding mode in which no information about expected binding site is provided.

Type	Compound	Binding Energy [kJ mol^{-1}]	Total Energy [kJ mol^{-1}]	E_{vdV} [kJ mol^{-1}][a]	E_{el} [kJ mol^{-1}][b]
Targeted	Glc-α	-28.05	-202.84	50.94	-253.78
	Glc-β	-28.07	-217.10	56.73	-273.83
	2-DG-α	-28.38	-207.19	14.71	-221.90
	2-DG-β	-28.38	-211.21	17.39	-228.60
	2-FG-α	-28.93	-196.05	11.04	-207.09
	2-FG-β	-29.00	-203.09	11.74	-214.83
	2,2-diFG-α	-29.10	-193.82	11.11	-204.93
	2,2-diFG-β	-28.88	-193.98	40.89	-234.87
	2-CG-α	-28.59	-149.21	71.45	-220.66
	2-CG-β	-28.72	-156.56	68.20	-224.76
	2-BG-α	-28.47	-92.28	65.56	-157.84
	2-BG-β	-28.34	-129.81	116.32	-246.13

Blind	Glc-α	-24.91	-152.93	-34.87	-118.06
	Glc-β	-26.62	-175.99	-37.46	-138.53
	2-DG-α	-26.98	-159.92	-13.67	-146.25
	2-DG-β	-26.56	-155.89	-12.81	-143.08
	2-FG-α	-27.44	-157.77	-11.73	-146.04
	2-FG-β	-24.58	-172.87	0.25	-173.12
	2,2-diFG-α	-26.52	-161.17	-2.52	-158.65
	2,2-diFG-β	-24.72	-158.42	-33.69	-124.73
	2-CG-α	-25.89	-162.56	-19.00	-143.56
	2-CG-β	-24.84	-160.06	-35.32	-124.74
	2-BG-α	-25.80	-153.63	-48.16	-105.46
	2-BG-β	-26.30	-171.23	-37.07	-134.16
	6P-Glc-α	-25.96	-247.25	17.83	-265.08
	6P-2DG-α	-25.17	-271.20	32.48	-303.67
	6P-2FG-α	-26.36	-273.78	-15.00	-258.78
	6P-2,2-diFG-α	-25.90	-260.97	35.25	-296.22
	6P-2CG-α	-26.85	-264.36	14.52	-278.88
	6P-2BG-α	-23.38	-238.68	8.09	-246.77

[a]Van der Waals contribution to the energy [b]electrostatic contribution to the energy

Table S6. Percentage contributions to the Hirshfeld surface area of each type of intermolecular contact for 2-DG and its analogs bound to HKII.

contact	2-DG-α	2-DG-β	2-FG-α	2-FG-β	2,2-diFG-α	2-diFG-β	2-CG-α	2-CG-β	2-BG-α	2-BG-β
H-H	48.3	49.7	42.9	42.2	37.5	36.2	40.2	40	39.7	39
H-O	46.7	45.6	42.7	43.7	40.6	41.8	41.2	41.7	39.9	41.3
H-C	2.0	2.0	1.9	1.9	1.9	1.9	1.6	1.8	1.5	1.4
H-N	1.2	1.2	1.3	1.2	1.2	1.2	1.0	1.2	1.0	1.1
C-O	0.5	0.5	0.5	0.5	0.5	0.5	0.4	0.4	0.5	0.5
O-O	0.8	0.5	0.8	0.5	0.6	0.5	0.8	0.4	1.0	0.8
O-N	0.5	0.5	0.5	0.5	0.5	0.4	0.5	0.5	0.5	0.4
H-F			7.6	8.0	11.9	14.1				
O-F			1.8	1.6	5.4	3.4				
H-Cl							9.9	11.0		
O-Cl							4.3	2.9		
H-Br									10.9	10.8
O-Br									4.9	4.6

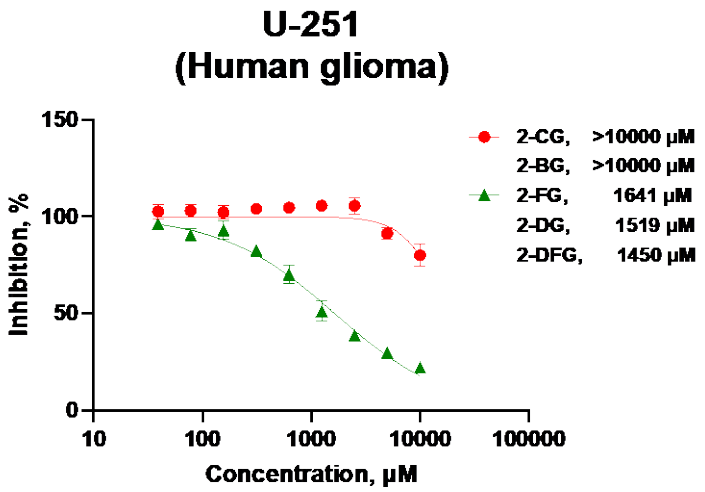

Figure S1. Viability of U-251 cells after 72 h treatment with various concentrations [0.5-10 mM] of 2-DG, 2-FG, 2,2-diFG (2-DFG), 2-CG and 2-BG.

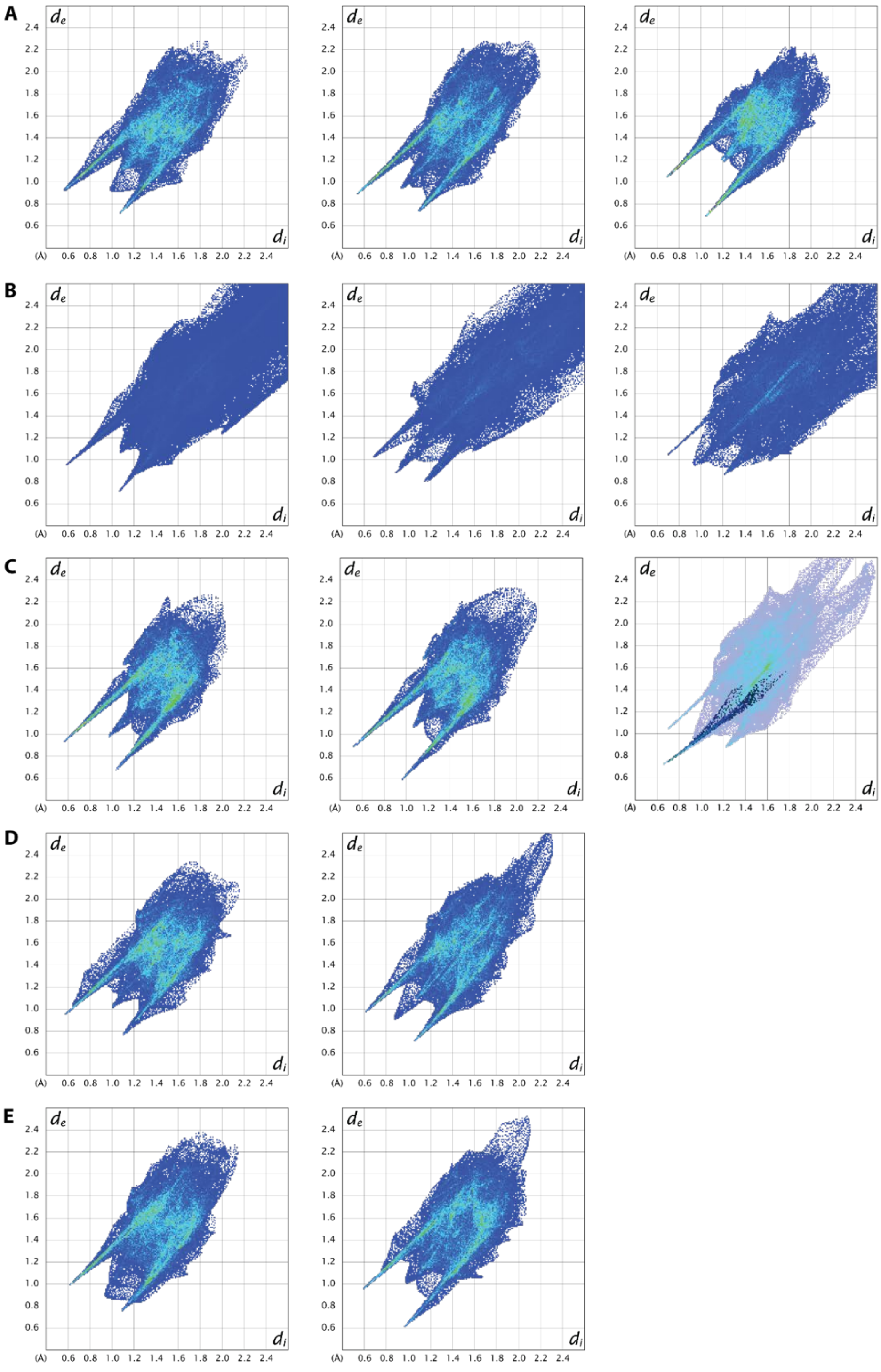

Figure. S2. Fingerprint plots for selected protein-ligand complexes relevant to the study. **A**. Comparison between glucose molecules in HKII binding cavities (N-terminal and C-terminal, left, middle) and glucose crystal. **B**. Fingerprints for HKII complexed with inhibitors 604, 603 and 62C (left, middle and right, respectively). **C**. Fingerprints of glucose molecules in N-terminal (left) and C-terminal (right) binding cavities of HKI, and pyranose part of 62C molecule binding to HKII (right, part belonging to covalent bond linking pyranose to the rest of 62C molecule is darkened). **D**. Fingerprints of glucose molecules in N-terminal (left) and C-terminal (middle) binding cavities of HKI complexed with mannose-6-phosphate. **E**. Fingerprints of glucose molecules in N-terminal (left) and C-terminal (middle) binding cavities of HKI complexed with 2-DG-6P.

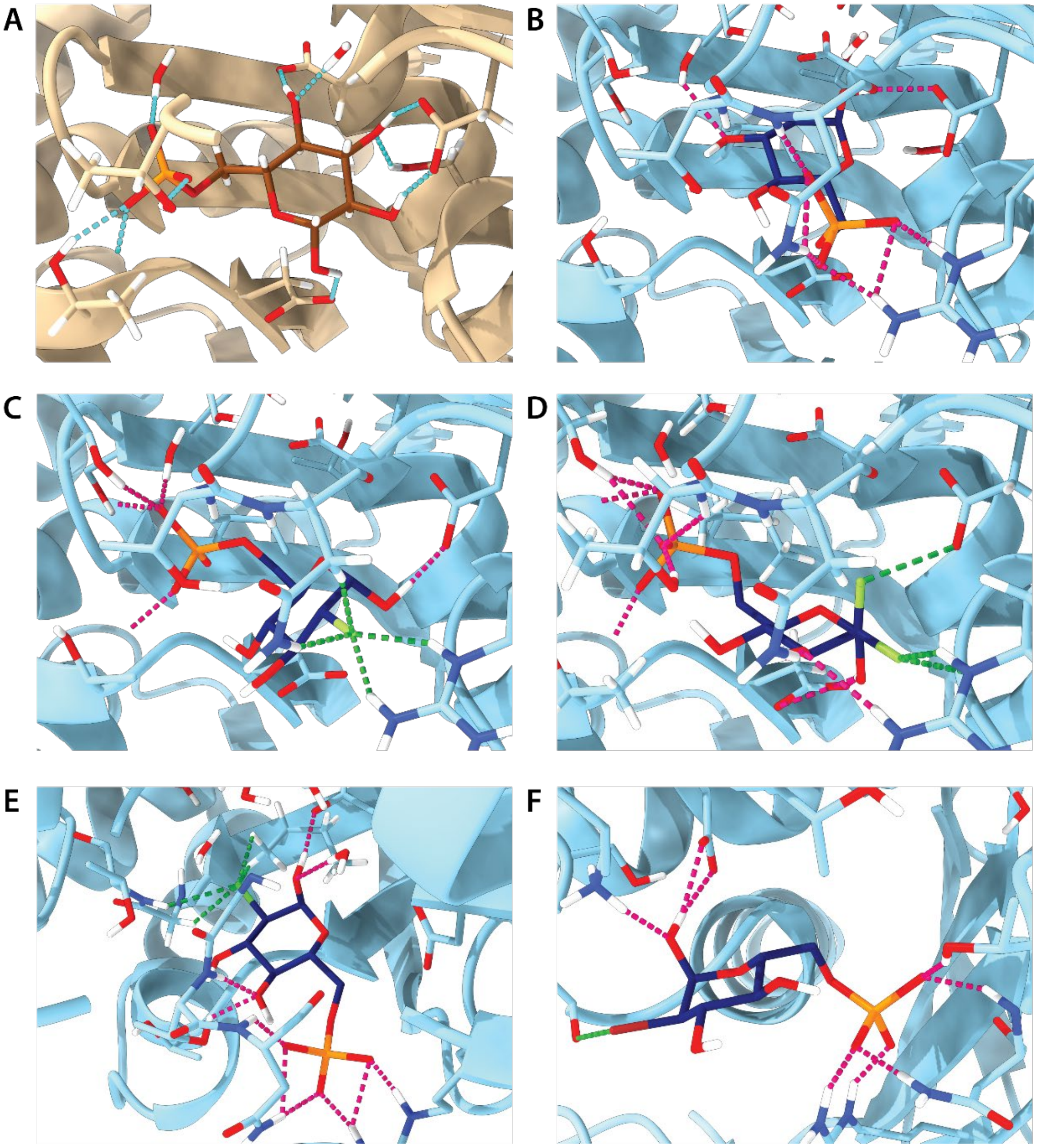

Figure S3. Results of molecular docking of 6-phosphates of glucose and its derivatives to HKII. In each panel most hydrogen atoms and amino acid residues were omitted for clarity. Typical H-bonds are depicted as pink dotted lines and close contacts involving a 2′ substituted moiety (H atom or halogen) are depicted as green dotted lines. If not mentioned otherwise only the α anomers are presented. (A) Binding of *Glc*-6P (experimental data from literature, PDB entry 2NTZ), typical H-bonds are depicted as cyan dotted lines; (B) Binding of 2-DG-6P and its derivatives: (C) 2-FG-6P, (D) 2′2′-diFG-6P, (E) 2-CG-6P and (F) 2-BG-6P.

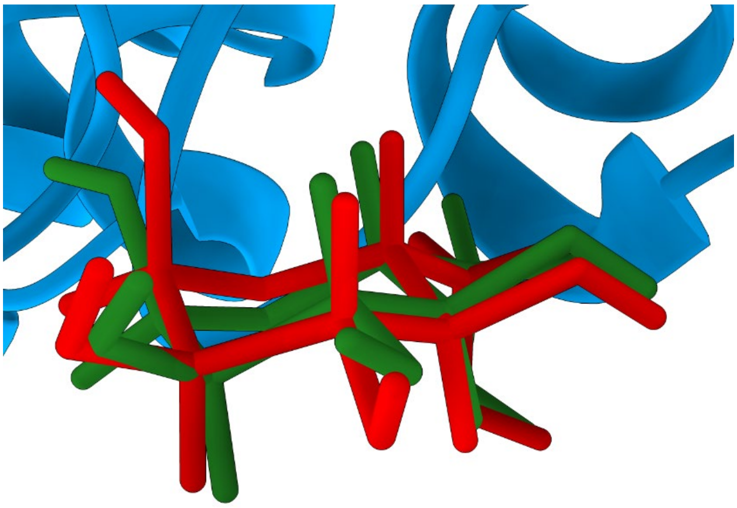

Figure S4. Testing of DockThor docking method. Superimposed glucose conformations from experimental structure (green) and molecular docking (red).
